# Exploring the antibiogram of soil isolates from an indian hospital precinct: link to antibiotic usage

**DOI:** 10.1186/s13104-023-06450-8

**Published:** 2023-08-15

**Authors:** Shalini Kunhikannan, Colleen J. Thomas, M. N. Sumana, Ashley E. Franks, Sumana Kumar, S. Nagarathna, Steve Petrovski, Anya E. Shindler

**Affiliations:** 1 Department of Microbiology, Anatomy, Physiology and Pharmacology, School of Agriculture, Biomedicine and Environment, La Trobe University, Bundoora, VIC 3086 Australia; 2 Department of Microbiology, JSS Medical College and Hospital, Mysuru, Karnataka India; 3 Centre for Cardiovascular Biology and Disease Research, School of Agriculture, Biomedicine and Environment, La Trobe University, Bundoora, VIC Australia; 4 Florey Institute of Neuroscience and Mental Health, Pre-clinical Critical Care Unit, University of Melbourne, Melbourne, VIC Australia; 5 Centre for Future Landscapes, La Trobe University, Bundoora, VIC Australia; 6 Department of Microbiology, Faculty of Life Sciences, JSS Academy of Higher Education and Research, Mysuru, Karnataka India; 7 Professor and Head, National Institute of Mental Health and Neurosciences, Bangalore, India

**Keywords:** Antibiotic resistance, Hospitals, *Pseudomonas*, Soil, Ticarcillin-clavulanic acid

## Abstract

**Objective:**

Hospitals serve as hotspots of antibiotic resistance. Despite several studies exploring antibiotic resistance in hospitals, none have explored the resistance profile of soil bacteria from a hospital precinct. This study examined and compared the antibiogram of the soil isolates from a hospital and its affiliated university precinct, to determine if antibiotic resistant bacteria were present closer to the hospital.

**Results:**

120 soil samples were collected from JSS Hospital and JSS University in Mysore, India across three consecutive seasons (monsoon, winter and summer). 366 isolates were randomly selected from culture. Antibiotic susceptibility testing was performed on 128 isolates of *Pseudomonas* (n = 73), *Acinetobacter* (n = 30), *Klebsiella* species (n = 15) and *Escherichia coli* (n = 10). *Pseudomonas* species exhibited the highest antibiotic resistance. Ticarcillin-clavulanic acid, an extended-spectrum carboxypenicillin antibiotic used to treat moderate-to-severe infections, ranked highest amongst the antibiotics to whom these isolates were resistant (n = 51 out of 73, 69.9%). Moreover, 56.8% (n = 29) were from the hospital and 43.1% (n = 22) were from the university precinct, indicating antibiotic resistant bacteria were closer to the hospital setting. This study highlights the effect of antibiotic usage in hospitals and the influence of anthropogenic activities in the hospital on the dissemination of antibiotic resistance into hospital precinct soil.

**Supplementary Information:**

The online version contains supplementary material available at 10.1186/s13104-023-06450-8.

## Introduction

Antibiotics use in hospitals contribute to the burden of antimicrobial resistance (AR). Recent evidence suggests lower-middle income countries like India, China and Pakistan are driving this phenomenon [1]. Increased usage of antibiotics by humans in day-day activities has led to an escalation of antibiotic resistance genes (ARG’s) [1]. As we recently highlighted, there are different environmental hotspots for ARG’s [2], but only minimal information regarding Measured environmental concentrations (MEC) of pharmaceuticals (including antibiotics) is available from the soil [3]. Metagenomic and network analysis of ARG abundances indicates an increase of ARG’s from anthropogenically ‘less disturbed’ to ‘highly disturbed’ environments, correlating with antibiotic usage in human and veterinary medicine [4]. However, it has been a challenge to determine the origin of ARG’s among environmental bacteria [5]. Improper sanitary practices and low sewer connectivity can be one of the factors leading to antibiotics entering the environment [6]. Anthropogenic activities together with environmental factors enhance the dissemination of ARG’s within nosocomial pathogens [7, 8] via horizontal gene transfer (HGT) [9, 10]. Although the global spread of antibiotic-resistant bacteria (ARB) and genes between water, soil, air and food is documented, the factors affecting the dissemination of resistance determinants is just beginning to unfold [11].

This study aimed to analyze if antibiotic usage in an Indian hospital had any effect on the ecology of the soil environment. To the authors’ knowledge, this is the first study to analyze the antibiotic resistance pattern among the soil isolates from a hospital precinct and determine whether the prevalence of ARB is higher closer to a hospital.

## Main text

## Results

A total of 120 soil samples were collected: 40 samples each during three different seasons (mainly monsoon, winter and summer) in Mysore, India. This study focused on Gram-negative organisms – *Pseudomonas*, *Acinetobacter*, *Klebsiella* and *Escherichia coli*, as they are well known to cause major infections in humans and have a higher capacity for uptake of resistance determinants from the environment. The soil samples were cultured on MacConkey agar and 366 randomly selected isolates were picked up from the culture. Among these, 128 isolates were obtained during the monsoon, 134 isolates during the winter and 104 during the summer. Further sub-cultures were performed on MacConkey agar to obtain pure cultures. The obtained isolates were confirmed by MALDI-TOF mass spectrometry. The MALDI-TOF analysis results of all 366 randomly selected isolates are provided in Supplementary Table S1. As our study focused on clinically relevant Gram-negative bacteria (*Pseudomonas*, *Acinetobacter*, *Klebsiella* and *Escherichia coli*), it was observed that 128 isolates belonged to this genus and they were further screened for Antimicrobial Susceptibility Testing (AST). Among these isolates, 60 (46.9%) were from the hospital precinct and 68 (53.1%) from the university precinct. The AST pattern of the soil isolates obtained from the hospital and university have been provided in Supplementary Table S2 and S3.

### Pseudomonas isolates

Out of the 128 isolates identified, 73 (57%) belonged to the *Pseudomonas* genus. The most common species was *Pseudomonas putida* (n = 40, 54.7%), followed by *P. mossellii* (n = 10, 13.7%), *P. aeruginosa* (n = 8, 10.9%), *P. mendocina* (n = 6, 8.2%), *P. alcaligenes* (n = 5, 6.8%), *P. stramineae* (n = 2, 2.7%), *P. stutzeri* (n = 1, 1.4%), while it was not possible to speciate one isolate (Table 1). The antibiotic resistance of each *Pseudomonas* strain isolated was determined using the Automated VITEK® 2 (Biomerieux) assay and the results of antibiotic resistance and sensitivity pattern of the *Pseudomonas* species are provided in Table 1. According to the CLSI Guidelines 2019 [12], *Pseudomonas* species are intrinsically resistant to tigecycline (TGC), and this was confirmed in our results (Table 1). In addition, the most resistance was observed to the β-lactams (βL) and β-lactams-β-lactamase (βL-βlactamase) inhibitor antibiotic combinations and the Folate pathway (FP) inhibitor antibiotics. Among the 73 strains of *Pseudomonas*, 69.9% (n = 51) were resistant to ticarcillin-clavulanic acid (TIC) and 64.4% (n = 47) were resistant to trimethoprim-sulphomethoxazole (COT) (Table 2). Among the class of Carbapenem antibiotics, 2.7% (n = 2) of Pseudomonas isolates were resistant to Imipenem (IMP), while 1.36% (n = 1) showed intermediate sensitivity to meropenem (MRP) (Table 2). All isolates (n = 73, 100%) were sensitive to amikacin (AK), ciprofloxacin (CIP), levofloxacin (LEV) (Table 2). Most of the isolates were sensitive to MRP (n = 71, 98.6%), piperacillin-tazobactam (PIT) (n = 71, 97.3%), ceftazidime (CAZ) (n = 71, 97.3%), cefeperazone (CEF) (n = 71, 97.3%) cefeparazone-sulbactam (SCF) (n = 71, 97.3%), IMP (n = 71, 97.3%), and gentamycin (GEN) (n = 71, 97.3%) (Table 2). Intermediate sensitivity was exhibited by one isolate each against MRP (n = 1, 1.4%), PIT (n = 1, 1.3%) and CAZ (n = 1, 1.4%) (Table 2).

**Table 1:** Species-wide distribution of *Pseudomonas*, *Acinetobacter*, *Escherichia coli* and *Klebsiella* species isolates from soil samples

		AMP	AMC	TIC	PIT	CXM / AXT	CXM	CTX	CAZ	SCF	CEF	CPM	DOR	IMP	MRP	ETP	AK	GEN	CIP	LEV	MIN	TGC	COT	CL	NIT	NA
***P. putida*** (n = 40), 54.70%	**S**	-	-	1	38	-	-	-	38	38	38	-	-	40	40	-	40	40	40	40	-	0	2	-	-	-
	**I**	-	-	0	1	-	-	-	1	0	0	-	-	0	0	-	0	0	0	0	-	0	0	-	-	-
	**R**	-	-	39	1	-	-	-	1	2	2	-	-	0	0	-	0	0	0	0	-	40	38	-	-	-
***P. mosselii*** (n = 10), 13.70%	**S**	-	-	0	10	-	-	-	10	10	10	-	-	8	10	-	10	10	10	10	-	4	3	-	-	-
	**I**	-	-	0	0	-	-	-	0	0	0	-	-	0	0	-	0	0	0	0	-	0	0	-	-	-
	**R**	-	-	10	0	-	-	-	0	0	0	-	-	2	0	-	0	0	0	0	-	6	7	-	-	-
***P. aeruginosa*** (n = 8), 10.90%	**S**	-	-	7	8	-	-	-	8	8	8	-	-	8	8	-	8	8	8	8	-	0	-	-	-	-
	**I**	-	-	0	0	-	-	-	0	0	0	-	-	0	0	-	0	0	0	0	-	0	-	-	-	-
	**R**	-	-	1	0	-	-	-	0	0	0	-	-	0	0	-	0	0	0	0	-	8	-	-	-	-
***P.mendocina*** (n = 6), 8.20%	**S**	-	-	6	6	-	-	-	6	6	6	-	-	6	6	-	6	6	6	6	-	0	6	-	-	-
	**I**	-	-	0	0	-	-	-	0	0	0	-	-	0	0	-	0	0	0	0	-	0	0	-	-	-
	**R**	-	-	0	0	-	-	-	0	0	0	-	-	0	0	-	0	0	0	0	-	6	0	-	-	-
***P. alcaligenes*** (n = 5), 6.80%	**S**	-	-	5	5	-	-	-	5	5	5	-	-	5	5	-	5	5	5	5	-	0	4	-	-	-
	**I**	-	-	0	0	-	-	-	0	0	0	-	-	0	0	-	0	0	0	0	-	0	0	-	-	-
	**R**	-	-	0	0	-	-	-	0	0	0	-	-	0	0	-	0	0	0	0	-	5	1	-	-	-
***P. stramineae*** (n = 2), 2.73%	**S**	-	-	2	2	-	-	-	2	2	2	-	-	2	2	-	2	0	2	2	-	0	2	-	-	-
	**I**	-	-	0	0	-	-	-	0	0	0	-	-	0	0	-	0	0	0	0	-	0	0	-	-	-
	**R**	-	-	0	0	-	-	-	0	0	0	-	-	0	0	-	0	2	0	0	-	2	0	-	-	-
***P. stutzeri*** (n-1), 1.36%	**S**	-	-	1	1	-	-	-	1	1	1	-	-	1	1	-	1	1	1	1	NA	0	1	-	-	-
	**I**	-	-	0	0	-	-	-	0	0	0	-	-	0	0	-	0	0	0	0	NA	0	0	-	-	-
	**R**	-	-	0	0	-	-	-	0	0	0	-	-	0	0	-	0	0	0	0	NA	1	0	-	-	-
***Pseudomonas*species** (n = 1), 1.36%	**S**	-	-	0	1	-	-	-	1	1	1	-	-	1	1	-	1	1	1	1	NA	0	0	-	-	-
	**I**	-	-	0	0	-	-	-	0	0	0	-	-	0	0	-	0	0	0	0	NA	0	0	-	-	-
	**R**	-	-	1	0	-	-	-	0	0	0	-	-	0	0	-	0	0	0	0	NA	1	1	-	-	-
***A pitti*** (n = 21), 70%	**S**	-	-	21	17	-	-	-	20	20	20	-	20	20	20	-	-	20	21	21	21	21	21	0	-	-
	**I**	-	-	0	2	-	-	-	0	1	0	-	0	0	0	-	-	0	0	0	0	0	0	21	-	-
	**R**	-	-	0	2	-	-	-	1	0	1	-	1	1	1	-	-	1	0	0	0	0	0	0	-	-
***A.radioresistans*** (n = 3), 10%	**S**	-	-	3	3	-	-	-	3	3	3	-	3	3	3	-	-	3	3	3	3	3	3	0	-	-
	**I**	-	-	0	0	-	-	-	0	0	0	-	0	0	0	-	-	0	0	0	0	0	0	3	-	-
	**R**	-	-	0	0	-	-	-	0	0	0	-	0	0	0	-	-	0	0	0	0	0	0	0	-	-
***A. calcoaceticus*** (n = 3), 10%	**S**	-	-	3	3	-	-	-	3	3	3	-	3	3	3	-	-	3	3	3	3	3	3	0	-	-
	**I**	-	-	0	0	-	-	-	0	0	0	-	0	0	0	-	-	0	0	0	0	0	0	3	-	-
	**R**	-	-	0	0	-	-	-	0	0	0	-	0	0	0	-	-	0	0	0	0	0	0	0	-	-
***A. junnii*** (n = 1), 3.3%	**S**	-	-	1	1	-	-	-	1	1	1	-	1	1	1	-	-	1	1	1	1	1	1	0	-	-
	**I**	-	-	0	0	-	-	-	0	0	0	-	0	0	0	-	-	0	0	0	0	0	0	1	-	-
	**R**	-	-	0	0	-	-	-	0	0	0	-	0	0	0	-	-	0	0	0	0	0	0	0	-	-
***A. gyllenberggii*** (n = 1) ), 3.3%	**S**	-	-	1	1	-	-	-	1	1	1	-	1	1	1	-	-	1	1	1	1	1	1	0	-	-
	**I**	-	-	0	0	-	-	-	0	0	0	-	0	0	0	-	-	0	0	0	0	0	0	1	-	-
	**R**	-	-	0	0	-	-	-	0	0	0	-	0	0	0	-	-	0	0	0	0	0	0	0	-	-
***A.lwoffii*** (n = 1) ), 3.3%		Terminated (could not perform AST – attempted twice)																								
***E. coli*** (n = 10), 100%	**S**	8	10	-	10	8	8	8	-	10		10	-	10	10	10	10	10	10	-	-	10	10	0	9	10
	**I**	0	0	-	0	0	0	0	-	0		0	-	0	0	0	0	0	0	-	-	0	0	10	0	0
	**R**	2	0	-	0	2	2	2	-	0		0	-	0	0	0	0	0	0	-	-	0	0	0	1	0
*** K.pneumoniae*** (n = 10), 66.6%	**S**	0	9	-	10	10	10	10	-	10		10	-	10	10	10	10	10	10	-	-	10	10	1	1	10
	**I**	0	0	-	0	0	0	0	-	0		0	-	0	0	0	0	0	0	-	-	0	0	9	9	0
	**R**	10	1	-	0	0	0	0	-	0		0	-	0	0	0	0	0	0	-	-	0	0	0	0	0
*** K.oxytoca*** (n = 4), 26.6%	**S**	0	3	-	4	4	4	4	-	4		4	-	4	4	4	4	4	4	-	-	4	4	3	3	4
	**I**	0	0	-	0	0	0	0	-	0		0	-	0	0	0	0	0	0	-	-	0	0	1	1	0
	**R**	4	1	-	0	0	0	0	-	0		0	-	0	0	0	0	0	0	-	-	0	0	0	0	0
*** K.varicola*** (n = 1), 6.67%	**S**	0	1	-	1	1	1	1	-	1		1	-	1	1	1	1	1	1	-	-	1	1	0	0	1
	**I**	0	0	-	0	0	0	0	-	0		0	-	0	0	0	0	0	0	-	-	0	0	1	1	0
	**R**	1	0	-	0	0	0	0	-	0		0	-	0	0	0	0	0	0	-	-	0	0	0	0	0

S, susceptible; I, intermediate; R, resistant. “-“ denotes not applicable. AMP, Ampicillin; AMC, Amoxyclav; TIC, Ticarcillin-clavulanic acid; PIT, Piperacillin tazobactam, CXM, Cefuroxime; CXM/AXT, Cefuroxime auxetil; CTX, Ceftriaxone; CAZ, Ceftazidime; SCF, Cefeperozone-sulbactam; CEF, Cefeperazone; CPM, Cefepime; DOR, Doripenem,; IMP, Imipenem; MRP, Meropenem; ETP, Ertapenem; AK, Amikacin; GEN, Gentamycin; CIP, Ciprofloxacin; LEV, Levofloxacin; MIN, Minocycline; TGC, Tigecycline; COT, Trimethoprim-sulphamethoxazole. CL, Colistin; NIT, Nitrofurantoin; NA, Nalidixic acid

**Table 2:** Antibiogram of *Pseudomonas*, *Acinetobacter*, and *Klebsiella* species isolated from soil samples

		βL	βL + βlase				βL			βL + βlase	βL		Carbapenems				Aminoglycosides		Quinolones			Tetracyclines		FA inhibitor	PM	NF
		AMP n, (%)	AMC n, (%)	TIC n, (%)	PIT n, (%)	CXM / AXT n, (%)	CXM n, (%)	CTX n, (%)	CAZ n, (%)	SCF n, (%)	CEF n, (%)	CPM n, (%)	DOR n, (%)	IMP n, (%)	MRP n, (%)	ETP n, (%)	AK n, (%)	GEN n, (%)	CIP n, (%)	LEV n, (%)	NA n, (%)	MIN n, (%)	TGC n, (%)	COT n, (%)	CL n, (%)	NIT n, (%)
***Pseudomonas*** **species (n = 73)**	**S**	-	-	22 (30.1)	71 (97.3)	-	-	-	71 (97.3)	71 (97.3)	71 (97.3)	-	-	71 (97.3)	72 (98.6)	-	73 (100)	71 (97.3)	73 (100)	73 (100)	-	-	0 (0)	18 (24.6)	-	-
	**I**	-	-	0 (0)	1 (1.36)	-	-	-	1 (1.36)	0 (0)	0 (0)	-	-	0 (0%)	1 (1.36)	-	0 (0)	0 (0)	0 (0)	0 (0)	-	-	0 (0)	0 (0)	-	-
	**R**	-	-	51 (69.9)	1 (1.4)	-	-	-	1 (1.36)	2 (2.7)	2 (2.7)	-	-	2 (2.7)	0 (0)	-	0 (0)	2 (2.7)	0 (0)	0 (0)	-	-	73 (100)	47 (64.4)	-	-
	**N/A**																							8 (11.0)		
***Acinetobacter*** **species** ***n = 30***	** S**	-	-	28 (93.3)	25 (83.3)	-	-	-	28 (93.3)	28 (93.3)	28 (93.3)	-	28 (93.3)	28 (93.3)	28 (93.3)	-	-	28 (93.3)	29 (96.6)	29 (96.6)	-	29 (96.6)	29 (96.6)	29 (96.6)	0 (0)	0 (0)
	**I**	-	-	0 (0)	0 (0)	-	-	-	0 (0)	0 (0)	0 (0)	-	0 (0)	0 (0)	0 (0)	0 (0)	-	0 (0)	0 (0)	0 (0)	-	0 (0)	0 (0)	0 (0)	29 (96.6)	-
	**R**	-	-	1 (3.3)	2 (6.6)	-	-	-	1 (3.3)	0	1 (3.3)	-	1 (3.3)	1 (3.3)	1 (3.3)	-	-	1 (3.3)	0 (0)	0 (0)	-	0 (0)	0 (0)	0 (0)	0 (0)	0 (0)
	**N/A**	-	-	1 (3.3)	1 (3.3)	-	-	-	1 (3.3)	1 (3.3)	1 (3.3)	-	1 (3.3)	1 (3.3)	1 (3.3)	-	-	1 (3.3)	1 (3.3)	1 (3.3)	-	1 (3.3)	1 (3.3)	1 (3.3)	1 (3.3)	-
***Klebsiella*** **species** ***(n = 15)***	**S**	0 (0)	13 (86.6)	-	15 (100)	15 (100)	15 (100)	15 (100)	-	1 (3.3)	-	15 (100)	-	15 (100)	15 (100)	15 (100)	15 (100)	15 (100)	15 (100)	-	15 (100)	-	15 (100)	15 (100)	0 (0)	0 (0)
	**I**	0 (0)	0 (0)	-	0 (0)	0 (0)	0 (0)	0 (0)	-	0 (0)	-	0 (0)	-	0 (0)	0 (0)	0 (0)	0 (0)	0 (0)	0 (0)	-	0 (0)	-	0 (0)	0 (0)	15 (100)	11 (73.3)
	**R**	15 (100)	2	-	0 (0)	0 (0)	0 (0)	0 (0)	-	0 (0)	-	0 (0)	-	0 (0)	0 (0)	0 (0)	0 (0)	0 (0)	0 (0)	-	0 (0)	-	0 (0)	0 (0)	4 (26.6)	4 (26.6)

S, susceptible; I, intermediate; R, resistant. “-“ denotes not applicable. AMP, Ampicillin; AMC, Amoxyclav; TIC, Ticarcillin-clavulanic acid; PIT, Piperacillin tazobactam, CXM, Cefuroxime; CXM/AXT, Cefuroxime auxetil; CTX, Ceftriaxone; CAZ, Ceftazidime; SCF, Cefeperozone-sulbactam; CEF, Cefeperazone; CPM, Cefepime; DOR, Doripenem,; IMP, Imipenem; MRP, Meropenem; ETP, Ertapenem; AK, Amikacin; GEN, Gentamycin; CIP, Ciprofloxacin; LEV, Levofloxacin; MIN, Minocycline; TGC, Tigecycline; COT, Trimethoprim-sulphamethoxazole. CL, Colistin; NIT, Nitrofurantoin; NA, Nalidixic acid

Among the 51 *Pseudomonas* soil isolates resistant to TIC, the majority (n = 29, 56.8%) were obtained from the hospital precinct. Among the 47 *Pseudomonas* isolates resistant to COT, the majority (n = 28, 59.6%) were also from the hospital setting. Two *Pseudomonas* isolates were resistant to IMP; one each came from the hospital and university. Intermediate resistance against MRP was exhibited by one isolate obtained from the hospital. GEN resistance was only observed in two isolates, from the university.

### Acinetobacter isolates

Out of the 128 isolates identified, 30 (23.4%) belonged to the *Acinetobacter* genus. The species of *Acinetobacter* identified were *A. pitti* (n = 21, 70%), *A. radioresistans* (n = 3, 10%), *A. calcoaceticus* (n = 3, 10%), *A. junnii* (n = 1, 3.3%), *A. gyllenberggii* (n = 1, 3.3%) and *A. lwoffii* (n = 1, 3.3%) (Table 1). Among the 30 strains of *Acinetobacter*, resistance was exhibited only by *Acinetobacter pitti* (Table 1). A. pitti isolates showed resistance against TIC (n = 1, 3.3%), PIT (n = 2, 6.7%), CAZ (n = 1, 3.3%), CEF (n = 1, 3.3%), SCF (n = 1, 3.3%), doripenem (DOR) (n = 1, 3.3%), IMP (n = 1, 3.3%), MRP (n = 1, 3.3%), and GEN (n = 1, 3.3%) (Table 2). Intermediate resistance was detected to PIT (n = 2, 6.7%) and SCF (n = 1, 3.3%) and colistin (n = 21, 70%) (Table 2). Among this was one strain, multi-drug resistant *Acinetobacter pitti*, which was from the university precinct. Among the 21 strains of *Acinetobacter pitti*, two exhibited resistance to PIT and two showed intermediate resistance (Table 1). Among these, one isolate exhibiting intermediate resistance was obtained from the hospital while the other resistant isolate and two intermediate isolates were from the university soil. One isolate of *A. lwoffii* was identified in our study (Table 1). We were unable to screen this isolate for antibiotic resistance since the VITEK® reaction was terminated upon two separate attempts. Apart from this isolate, all other isolates showed 100% sensitivity against LEV, MIN, TGC and COT. The antibiogram of different *Acinetobacter* isolates is summarized in Table 2.

### Escherichia coli isolates

Out of the 128 isolates identified, 10 were *Escherichia coli* strains. As highlighted in Table 1, resistance was observed against ampicillin (AMP) (n = 2, 20%), cefuroxime (CXM) (n = 2, 20%), cefuroxime-auxetil (CFM-AXT) (n = 2, 20%), ceftriaxone (CTX) (n = 2, 20%) and nitrofurantoin (NIT) (n = 1, 10%). All the isolates were sensitive to Amoxyclav (AMC), PIT, SCF, cefepime (CPM), ertapenem (ETP), MRP, AK, GEN, NA, CIP, TGC, NIT, COT (Table 1).

### Klebsiella isolates

Out of the 128 isolates identified, 15 belonged to the *Klebsiella* genus. The main species identified were *K. pneumoniae* (n = 10, 66.7%), *K. oxytoca* (n = 4, 26.6%) and *K. varicola* (1, 6.7%) (Table 1). Two *Klebsiella* species isolates (*K. pneumoniae*, *K. oxytoca*) were resistant to AMC (Table 1). Both of these isolates were from the university. Table 2 summarizes the antibiogram of all the *Klebsiella* species identified. Although NIT is normally not the drug of choice for *Klebsiella* infections, four isolates were resistant to NIT while 11 showed intermediate resistance. All isolates were intrinsically resistant to AMP (Table 2).

### AST of isolates obtained from patients at the hospital

#### Clinical Pseudomonas isolates

Data from the hospital records contained *Pseudomonas* (n = 780) during the study period. This included 102 isolates of Multidrug Resistant (MDR) *Pseudomonas aeruginosa* and 659 strains of *Pseudomonas aeruginosa*. The other species were *P. putida* (n = 8), *P. luteola* (n = 6), *P. fluorescens* (n = 3) and *P. stutzeri* (n = 2) (Table 3). Among the *Pseudomonas* species, resistance was observed against all the classes of antibiotics. Most of these isolates obtained in the hospital (clinical isolates) were *Pseudomonas aeruginosa* and the highest number of isolates were resistant to TIC (n = 209, 32%) and LEV (n = 185, 28%). The highest resistance exhibited by *P. putida* was against ceftazidime, TIC, MIN and Aztreoname (AZ). *P. luteola* displayed resistance to most antibiotics, with all the isolates resistant to cefepime, PIT, MRP and CIP (Table 3). All three isolates of *P. fluorescens* were resistant to TIC and MIN. Among the two isolates of *P. stutzeri*, both isolates were resistant to MIN while the other isolate was resistant to the βL and βL-βlactamase inhibitor combinations, aminoglycosides and quinolones (Table 3).

**Table 3:** Resistance exhibited by clinical isolates of *Pseudomonas* and *Acinetobacter* species against the major antibiotics

	CAZ	SCF	CPM	PIT	TIC	DOR	IMP	MRP	CIP	LEV	GEN	AK	TGC	MIN	COT	CL	AZ
***P. aeruginosa*** **(MDR)** N = 102 R, n (%)	90 (88.2%)	82 (80.4%)	92 (90.2%)	62 (60.8%)	82 (80.4%)	86 (84.3%)	95 (93.1%)	90 (88.2%)	96 (94.1%)	96 (94.1%)	91 (89.2%)	96 (94.1%)	94 (92.2%)	-	-	1 (1%)	32 (31.3%)
***P. aeruginosa*** N = 659 R, n (%)	153 (23.2%)	98 (14.9%)	111 (16.8%)	105 (16%)	209 (31.7%)	111 (16.8%)	153 (23.2)	116 (17.6%)	168 (25.5%)	185 (28.1%)	135 (20.5%)	116 (17.6%)	575 (87.3%)	-	-	24 (3.6%)	165 (25%)
***P. putida*** N = 8 R, n (%)	8 (100%)	3 (38%)	2 (25%)	2 (25%)	6 (75%)	-	2 (25%)	1 (13%)	4 (50%)	5 (63%)	2 (25%)	1 (13%)	8 (100%)	8 (100%)	6 (75%)	-	4 (50%)
***P. luteola*** N = 6 R, n (%)	5 (83%)	5 (83%)	6 (100%)	6 (100%)	5 (83%)	-	5 (83%)	6 (100%)	6 (100%)	2 (33.3%)	4 (67%)	3 (50%)	-	1 (16%)	4 (67%)	-	1 (16%)
***P. fluorescens*** N = 3 R, n (%)	2 (66.6%%)	1 (33.3%)	2 (66.6%%)	1 (33.3%)	3 (100%)	-	1 (33.3%)	1 (33.3%)	1 (33.3%)	1 (33.3%)	1 (33.3%)	2 (66.6%%)	3 (100%)	3 (100%)	3 (100%)	-	2 (66.6%%)
***P. stutzeri*** N = 2 R, n (%)	1 (50%)	1 (50%)	1 (50%)	1 (50%)	0 (0%)	-	0 (0%)	0 (0%)	1 (50%)	1 (50%)	1 (50%)	1 (50%)	2 (100%)	2 (100%)	1 (50%)	-	1 (50%)
***Acinetobacter baumannii*** **(MDR)** N = 315 R, n (%)	286 (90.8%)	287 (91.1%)	307 (97.5%)	307 (97.5%)	282 (89.5%)	282 (89.5%)	307 (97.5%)	305 (97%)	307 (97.5%)	231 (73.3%)	241 (76.5%)	-	4 (1.3%)	136 (43.2%)	238 (75.6%)	1 (0.3%)	-
***Acinetobacter baumannii*** N = 298 R, n (%)	206 (69.1%)	182 (61%)	234 (78.5%)	223 (74.8%)	214 (71.8%)	197 (66.1%)	216 (72.5%)	214 (71.8%)	216 (74.5%)	118 (39.6%)	128 (43%)	-	2 (0.7%)	63 (21.1%)	153 (51.3%)	10 (3.4%)	-
***Acinetobacter baumannii*complex** N = 61 R, n (%)	47 (77%)	43 (70.5%)	53 (86.9%)	52 (85.2%)	46 (75.4%)	47 (77%)	52 (85.2%)	52 (85.2%)	53 (86.9%)	36 (59%)	42 (68.9%)	-	0 (0%)	14 (22.9%)	43 (70.5%)	0 (0%)	-
***Acinetobacter lwoffii*** N = 29 R, n (%)	13 (44.8%)	8 (27.6%)	14 (48.3%)	11 (38%)	11 (38%)	11 (38%)	10 (34.5%)	13 (44.8%)	14 (48.3%)	8 (27.6%)	12 (41.4%)	7 (24.1%)	1 (3.4%)	1 (3.4%)	10 (34.5%)	2 (6.9%)	-
***Acinetobacter haemolyticus*** N = 8 R, n (%)	2 (25%)	1 (12.5%)	2 (25%)	2 (25%)	2 (25%)	2 (25%)	2 (25%)	2 (25%)	3 (37.5%)	2 (25%)	2 (25%)	1 (12.5%)	0 (0%)	2 (25%)	4 (50%)	0 (0%)	-
***Acinetobacter junii*** N = 2 R, n (%)	1 (50%)	0 (0%)	1 (50%)	1 (50%)	0 (0%)	1 (50%)	0 (0%)	1 (50%)	0 (0%)	0 (0%)	1 (50%)	0 (0%)	0 (0%)	0 (0%)	0 (0%)	0 (0%)	-

S, susceptible; I, intermediate; R, resistant. CAZ, Ceftazidime; SCF, Cefeperozone-sulbactam; CPM, Cefepime; PIT, Piperacillin tazobactam, TIC, Ticarcillin-clavulanic acid; DOR, Doripenem,; IMP, Imipenem; MRP, Meropenem; CIP, Ciprofloxacin; LEV, Levofloxacin; GEN, Gentamycin; AK, Amikacin; TGC, Tigecycline; MIN, Minocycline; COT, Trimethoprim-sulphamethoxazole. CL, Colistin; AZ, Aztreonam

#### Clinical Acinetobacter isolates

Data from the hospital records contained *Acinetobacter* (n = 713) during the study period. The main species included *A. baumannii* (n = 613), *A. baumannii* complex (n = 61), *A. lwoffii* (n = 29), *A. haemolyticus* (n = 8), *A. junnii* (n = 2) (Table 3). The lowest resistance was observed against TGC. *A. baumannii* isolates exhibited major resistance to CPM and PIT followed by IMP, MRP and CIP (Table 3). CPM, IMP, MRP and CIP were the major antibiotics against which *A. baumannii* complex exhibited resistance (Table 3). *A. lwoffii* was resistant to the major βL and βL-βlactamase inhibitor combinations, aminoglycosides and quinolones. Antibiotic resistance was comparatively lower in *A. haemolyticus* and *A. junnii* isolates (Table 3).

## Discussion

The significance of analyzing the antibiotic resistance profile of soil isolates lies in revealing possible links of transfer of antibiotics from hospitals to the soil. Our study identified soil bacteria within the precincts of a hospital and a university located several kilometers apart. Based on our AST results, the highest resistance was observed among the *Pseudomonas* isolates from the hospital precinct against TIC, followed by COT. IMP resistance was observed among two isolates, one each from the hospital and university. Although IMP resistance was comparatively smaller, the significance of this finding cannot be underestimated as carbapenems are the ‘last line’ of drugs used to treat *Pseudomonas* infections [13], and the presence of these carbapenem-resistant (or I) isolates among soil isolates obtained in our study may be suggestive of a HGT event.

Previous analysis of clinical and environmental isolates of *P. aeruginosa* in Iran [14] showed highest resistance to TIC (55%). This was similar to our study, where the highest resistance was obtained against TIC. Hospital wastewater treatment plants are considered an important source of multi-drug resistant bacteria. A previous study in a hospital treatment plant in Brazil revealed that *Pseudomonas* isolates exhibited high resistance to AZ (62.9%), followed by TIC (33.3%) and CPM (22.2%) [15]. Among these, 22.2% were classified as multi-drug resistant. These findings illustrate the significant role of hospitals in the spread of AR to the surrounding environment. *Pseudomonas* species isolated from wastewater and its impacted marine zone previously, exhibited resistance against TIC and other antibiotics [16]. In Algeria, the antibiogram of Gram-negative bacteria identified from different vegetable sources revealed *Pseudomonas aeruginosa* to exhibit 92.9% resistance to TIC [17]. A previous study comparing samples collected in a market environment, clinical setting and poultry farms in Ghana provided evidence of genetic relatedness between environmental and clinical strains, which suggests an exchange of ARB between patients and environment [18]. Our study findings adds to this proposition. Comparison of our results from the soil to the clinical isolates obtained from the same hospital showed that the highest resistance observed in the soil isolates (TIC) was similar to the antibiotic against which most of the clinical isolates of *Pseudomonas aeruginosa* exhibited resistance. This could be suggestive of a strong link between the antibiotic usage in the hospitals and its dissemination to the surrounding soil. The disposal of raw sewage and occurrence of leaks from sewerage and septic systems contributes to a greater chance of diffuse pollution in lower-middle income countries [6], and this could be a potential reason for higher resistance observed in our study among *Pseudomonas* species closer to the hospitals. Hence, it is suggestive that significant anthropogenic activities in the hospital precinct, including sewage treatment plants, affect the surrounding soil.

Different regulatory frameworks, the healthcare facilities, population and local demographics, proximity of a pharmaceutical manufacturing, connectivity to wastewater treatment systems and ecology of the environment also control the concentration of pharmaceuticals and selection of resistance in an environment [6]. All these factors together favor the uptake of ARG’s from the soil. Considering that immune-compromised patients visit hospitals, the presence of any putative Gram-negative human pathogens capable of causing disease poses a risk of encountering humans, mobilizing into the human microbiome and causing disease. AR is a growing concern and its presence in an environment comprising more immunocompromised patients like clinical settings or hospitals adds on to the risk of development of multidrug resistant organisms.

To conclude, this study provides definitive evidence of antibiotic resistance in soil closer to the hospital settings and sheds light on a significant role of hospitals in the dissemination of AR. Further studies with a greater sample size and an antibiotic resistance profile of soil and clinical bacteria would provide further information on the possible links of transmission from hospitals to soil.

## Materials and methods

### Sample collection and setting

**Fig. 1:**
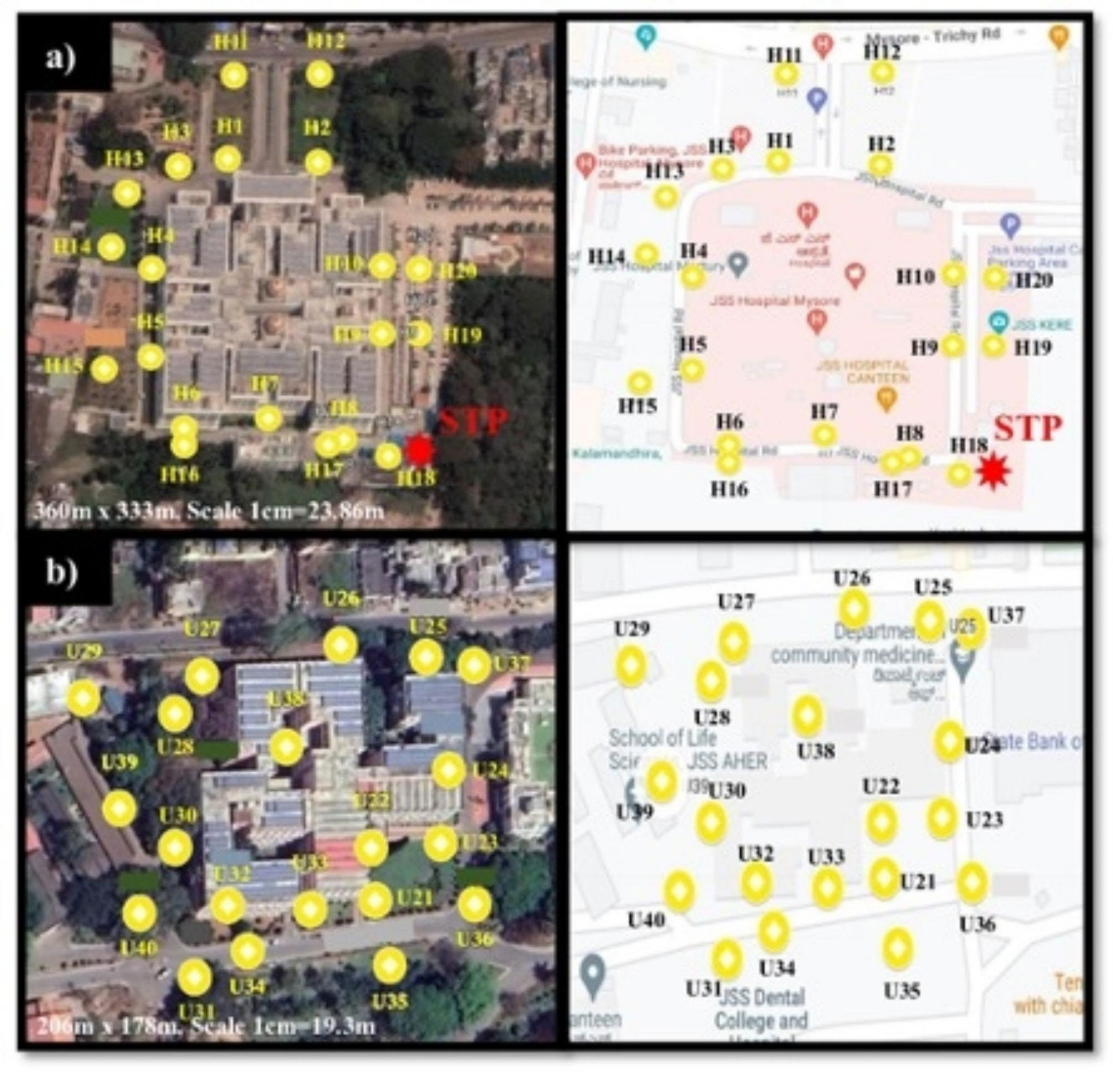
Location of soil sampling sites at JSS Hospital and JSS University in Mysore, India. (**a**) There were twenty soil sampling sites surrounding the hospital precinct, namely H1-H20. A sewage treatment plant (STP) was close to the sites - H8, H17, H18, H9 and H19. (**b**) There were also twenty soil sampling sites at the university, namely U21-U40. JSS, Jagadguru Sri Shivarathreeshwara. Adapted from Google maps.

Soil sampling sites for this study were the hospital precinct of a major 1800-bed hospital (Jagadguru Sri Shivarathreeshwara (JSS) Hospital) and the grounds of its affiliated university (JSS Academy of Higher Education and Research building) in Mysore (population of 1.21 million), India. The hospital and university precincts were situated ~6 km apart. Soil samples were collected from both precincts between June 2020 and May 2021. Soil samples were collected from 20 spots each, around the hospital (Fig. 1a) and University (Fig. 1b). Using sterile gloves, ~1 g of soil was collected from a patch 5 cm wide, along the four cardinal directions and placed into a sterile Nasco soil sampling bag. The samples were collected from all four cardinal directions and pooled to obtain samples from Site 1. In this way, a total of 4 g of soil was obtained from each sampling point. The same procedure was repeated to obtain samples from other sampling spots.

### Identification and antibiotic sensitivity testing

A gram (1 g) of soil was suspended in 10 ml saline (0.85% NaCl) and incubated for at room temperature for 10 min. Each of the soil samples were cultured by Spread plate technique on MacConkey agar plates (37 °C for 18–24 h). The culture plates were checked for any bacterial growth the next day. The plates were also incubated two more days at the same temperature to identify any slow growing bacteria in the soil. The obtained isolates were further sub-cultured to obtain pure cultures. This study focused on gram negative bacteria, mainly *Pseudomonas spp., Acinetobacter spp., Klebsiella spp., Escherichia coli*. Identification of the obtained isolates was performed using Matrix Assisted Laser Desorption Ionization-Time of Flight (MALDI-TOF) mass spectrometry (VITEK® MS V3.0) technique using the database VITEK® MS Knowledge Base V3.2. Organisms confirmed and identified as *Pseudomonas* species, *Acinetobacter* species, *Klebsiella* species, and *Escherichia coli*, were screened for AST. AST was performed using Automated VITEK® 2S00 (VITEK® 2 Systems, Version 9.0). A quality check was performed for every batch. In the case of Enterobacteriaceae family, *Escherichia coli (*ATCC 25,922) was used, while in case of *Pseudomonas* and *Acinetobacter*, *Pseudomonas* aeruginosa (ATCC 27,853) was used. AST was performed using AST-N280 (Enterobacteriaceae) and AST-N281 (*Pseudomonas* and *Acinetobacter*) cards in Automated VITEK® 2. The results were interpreted as Sensitive (S), Intermediate (I) and Resistant (R) based on the CLSI Guidelines 2019 [12].

Corresponding to the soil sampling times for this study from the hospital precinct (June 2020 to May 2021), the AST results of clinical isolates obtained from the hospital patients were examined for comparison. This data was extracted from the cloud-based Automated VITEK® 2S00 (VITEK® 2 Systems, Version 9.0) of JSS Hospital. The hospital data contained the results of analysis of clinical samples obtained from a range of samples including blood, urine and other body fluids. In brief, samples were cultured on MacConkey agar. The isolates obtained which were further sub-cultured to obtain pure colonies, followed by AST. These results were entered into the hospital database. Only data containing the AST of *Pseudomonas* and *Acinetobacter* species during the study period were extracted, as this study focused on Gram negative bacteria, and these were the prominent species identified in our study.

### Limitations

This study was performed on randomly selected isolates obtained by culture and does not provide the prevalence of Gram-negative bacteria in the soil. Antibiotic resistance gene profiling would provide a deeper understanding on the resistance genes in the human pathogens prevalent around a hospital.

### Electronic supplementary material

Below is the link to the electronic supplementary material.

Supplementary Material 1: Summary of the MALDI-TOF analysis and AST patterns for Pseudomonas, Acintobacter, Klebsiella, and Eschericha coli isolates

## Data Availability

All data generated and analyzed during the current study are available from the corresponding author on reasonable request.

## Abbreviations

AMC: Amoxyclav
AMP: Ampicillin
AMR: Antimicrobial resistance
ARB: Antibiotic-resistant bacteria
ARG: Antibiotic resistance gene
AR: Antimicrobial resistance
AZ: Aztreonam
CAZ: Ceftazidime
CEF: Cefeperazone
CIP: Ciprofloxacin
CL: Colistin
COT: Trimethoprim-sulphamethoxazole
CPM: Cefepime
CXM: Cefuroxime
CXM/AXT: Cefuroxime auxetil
CTX: Ceftriaxone
DOR: Doripenem
ETP: Ertapenem
GEN: Gentamycine
HGT: Horizontal gene transfer
IMP: Imipenem
LEV: Levofloxacin
MIN: Minocycline
MEC: Measured environmental concentrations
MRP: Meropenem
NA: Nalidixic acid
NIT: Nitrofurantoin
PIT: Piperacillin tazobactam
SCF: Cefeperozone-sulbactam
TGC: Tigecycline
TIC: Ticarcillin-clavulanic acid
