# Supplementary material for: Exploring the antibiogram of soil isolates from an indian hospital precinct: link to antibiotic usage
Source: BMC Res Notes. 2023 Aug 15;16:173. doi: 10.1186/s13104-023-06450-8 (PMC10428574; doi:10.1186/s13104-023-06450-8)
Supplement: Supplementary file 1 — Supplementary Material 1: Summary of the MALDI-TOF analysis and AST patterns for Pseudomonas, Acintobacter, Klebsiella, and Eschericha coli isolates [file 13104_2023_6450_MOESM1_ESM.docx]

# **Supplementary Information**

Supplementary Table 1

**Table 1:** **Summary of MALDI-TOF analysis of soil isolates from JSS Hospital and JSS University**.

| **Sampling spot** | **Sample ID for Culture isolates** | **Organism Identification by MALDI - TOF** |
| --- | --- | --- |
| SM/H/SEP20/01 | SM/H/SEP20/1.1 | *Pseudomonas putida* |
| SM/H/SEP20/01 | SM/H/SEP20/1.2 | Unidentified |
| SM/H/SEP20/01 | SM/H/SEP20/1.3 | *Stenotrophomonas maltophilia* |
| SM/H/SEP20/02 | SM/H/SEP20/2.1 | *Klebsiella pneumoniae* |
| SM/H/SEP20/02 | SM/H/SEP20/2.2 | *Pseudomonas mosselii* |
| SM/H/SEP20/02 | SM/H/SEP20/2.3 | *Pseudomonas putida* |
| SM/H/SEP20/03 | SM/H/SEP20/3.1 | *Klebsiella pneumoniae* |
| SM/H/SEP20/03 | SM/H/SEP20/3.2 | *Escherichia coli* |
| SM/H/SEP20/03 | SM/H/SEP20/3.3 | *Pseudomonas putida* |
| SM/H/SEP20/03 | SM/H/SEP20/3.4 | *Enterobacter kobei* |
| SM/H/SEP20/04 | SM/H/SEP20/4.1 | *Escherichia coli* |
| SM/H/SEP20/04 | SM/H/SEP20/4.2 | *Aeromonas punctata* |
| SM/H/SEP20/04 | SM/H/SEP20/4.3 | *Klebsiella pneumoniae* |
| SM/H/SEP20/04 | SM/H/SEP20/4.4 | *Pseudomonas putida* |
| SM/H/SEP20/05 | SM/H/SEP20/5.1 | *Acinetobacter pitti* |
| SM/H/SEP20/05 | SM/H/SEP20/5.2 | *Pseudomonas putida* |
| SM/H/AUG20/06 | SM/H/AUG20/6.1 | Unidentified |
| SM/H/AUG20/06 | SM/H/AUG20/6.2 | *Pantoea dispersa* |
| SM/H/AUG20/06 | SM/H/AUG20/6.3 | *Staphylococcus xylosus* |
| SM/H/AUG20/06 | SM/H/AUG20/6.4 | *Staphylococcus sciuri* |
| SM/H/AUG20/07 | SM/H/AUG20/7.1 | *Cupriavidus necator* |
| SM/H/AUG20/07 | SM/H/AUG20/7.2 | *Pseudomonas alkaligenes* |
| SM/H/AUG20/07 | SM/H/AUG20/7.3 | Unidentified |
| SM/H/AUG20/08 | SM/H/AUG20/8.1 | *Pseudomonas putida* |
| SM/H/AUG20/08 | SM/H/AUG20/8.2 | Unidentified |
| SM/H/SEP20/09 | SM/H/SEP20/9.1 | *Escherichia coli* |
| SM/H/SEP20/09 | SM/H/SEP20/9.2 | *Klebsiella varicola* |
| SM/H/SEP20/09 | SM/H/SEP20/9.3 | Unidentified |
| SM/H/SEP20/10 | SM/H/SEP20/10.1 | *Klebsiella pneumoniae* |
| SM/H/SEP20/10 | SM/H/SEP20/10.2 | *Pseudomonas mosselii* |
| SM/H/SEP20/10 | SM/H/SEP20/10.3 | *Acinetobacter pitti* |
| SM/H/SEP20/11 | SM/H/SEP20/11.1 | Unidentified |
| SM/H/SEP20/11 | SM/H/SEP20/11.2 | *Pseudomonas mosselii* |
| SM/H/SEP20/11 | SM/H/SEP20/11.3 | *Escherichia coli* |
| SM/H/SEP20/11 | SM/H/SEP20/11.4 | *Acinetobacter pitti* |
| SM/H/SEP20/12 | SM/H/SEP20/12.1 | *Pseudomonas putida* |
| SM/H/SEP20/12 | SM/H/SEP20/12.2 | *Enterobacter cloacae* |
| SM/H/SEP20/12 | SM/H/SEP20/12.3 | *Acinetobacter pitti* |
| SM/H/SEP20/12 | SM/H/SEP20/12.4 | Unidentified |
| SM/H/SEP20/13 | SM/H/SEP20/13.1 | *Enterobacter hormacheii* |
| SM/H/SEP20/13 | SM/H/SEP20/13.2 | *Klebsiella pneumoniae* |
| SM/H/SEP20/13 | SM/H/SEP20/13.3 | *Pseudomonas putida* |
| SM/H/SEP20/13 | SM/H/SEP20/13.4 | *Pseudomonas putida* |
| SM/H/SEP20/14 | SM/H/SEP20/14.1 | *Cupriavidus necator* |
| SM/H/SEP20/14 | SM/H/SEP20/14.2 | *Pseudomonas mosselii* |
| SM/H/SEP20/14 | SM/H/SEP20/14.3 | *Enterobacter hormacheii* |
| SM/H/SEP20/15 | SM/H/SEP20/15.1 | Unidentified |
| SM/H/SEP20/15 | SM/H/SEP20/15.2 | *Acinetobacter pitti* |
| SM/H/AUG20/16 | SM/H/AUG20/16.1 | *Cupriavidus necator* |
| SM/H/AUG20/16 | SM/H/AUG20/16.2 | *Pseudescherichia valneris* |
| SM/H/AUG20/16 | SM/H/AUG20/16.3 | *Leclercia adecarboxylata* |
| SM/H/AUG20/17 | SM/H/AUG20/17.1 | *Pseudomonas putida* |
| SM/H/AUG20/17 | SM/H/AUG20/17.2 | Unidentified |
| SM/H/AUG20/17 | SM/H/AUG20/17.3 | *Acinetobacter pitti* |
| SM/H/AUG20/18 | SM/H/AUG20/18.1 | *Acinetobacter pitti* |
| SM/H/AUG20/18 | SM/H/AUG20/18.2 | *Pseudomonas putida* |
| SM/H/AUG20/18 | SM/H/AUG20/18.3 | *Cupriavidus necator* |
| SM/H/SEP20/19 | SM/H/SEP20/19.1 | *Enterobacter chromaechiae* |
| SM/H/SEP20/19 | SM/H/SEP20/19.2 | *Klebsiella pneumoniae* |
| SM/H/SEP20/19 | SM/H/SEP20/19.3 | *Acinetobacter pitti* |
| SM/H/SEP20/19 | SM/H/SEP20/19.4 | Unidentified |
| SM/H/SEP20/20 | SM/H/SEP20/20.1 | *Pseudomonas putida* |
| SM/H/SEP20/20 | SM/H/SEP20/20.2 | *Enterobacter absuriae* |
| SM/H/SEP20/20 | SM/H/SEP20/20.3 | *Acinetobacter pitti* |
| SM/U/SEP20/21 | SM/U/SEP20/21.1 | *Enterobacter kobei* |
| SM/U/SEP20/21 | SM/U/SEP20/21.2 | Unidentified |
| SM/U/SEP20/21 | SM/U/SEP20/21.3 | *Acinetobacter pitti* |
| SM/U/SEP20/21 | SM/U/SEP20/21.4 | Unidentified |
| SM/U/SEP20/22 | SM/U/SEP20/22.1 | *Pseudomonas putida* |
| SM/U/SEP20/22 | SM/U/SEP20/22.2 | Unidentified |
| SM/U/SEP20/22 | SM/U/SEP20/22.3 | Unidentified |
| SM/U/SEP20/23 | SM/U/SEP20/23.1 | *Pseudomonas putida* |
| SM/U/SEP20/23 | SM/U/SEP20/23.2 | *Aeromonas hydrophila* |
| SM/U/SEP20/23 | SM/U/SEP20/23.3 | Unidentified |
| SM/U/SEP20/24 | SM/U/SEP20/24.1 | *Cupriavidus necator* |
| SM/U/SEP20/24 | SM/U/SEP20/24.2 | *Enterobacter kobei* |
| SM/U/SEP20/24 | SM/U/SEP20/24.3 | Unidentified |
| SM/U/SEP20/25 | SM/U/SEP20/25.1 | *Cupriavidus necator* |
| SM/U/SEP20/25 | SM/U/SEP20/25.2 | *Pseudomonas putida* |
| SM/U/SEP20/26 | SM/U/SEP20/26.1 | Unidentified |
| SM/U/SEP20/26 | SM/U/SEP20/26.2 | *Pseudomonas putida* |
| SM/U/SEP20/26 | SM/U/SEP20/26.3 | Unidentified |
| SM/U/SEP20/27 | SM/U/SEP20/27.1 | Unidentified |
| SM/U/SEP20/27 | SM/U/SEP20/27.2 | *Enterobacter hormaechei* |
| SM/U/SEP20/28 | SM/U/SEP20/28.1 | *Pseudomonas putida* |
| SM/U/SEP20/28 | SM/U/SEP20/28.2 | Unidentified |
| SM/U/SEP20/28 | SM/U/SEP20/28.3 | Unidentified |
| SM/U/SEP20/29 | SM/U/SEP20/29.1 | Unidentified |
| SM/U/SEP20/29 | SM/U/SEP20/29.2 | Unidentified |
| SM/U/SEP20/30 | SM/U/SEP20/30.1 | *Klebsiella oxytoca* |
| SM/U/SEP20/30 | SM/U/SEP20/30.2 | Unidentified |
| SM/U/SEP20/30 | SM/U/SEP20/30.3 | Unidentified |
| SM/U/SEP20/30 | SM/U/SEP20/30.4 | *Escherichia coli* |
| SM/U/SEP20/31 | SM/U/SEP20/31.1 | *Pseudomonas putida* |
| SM/U/SEP20/31 | SM/U/SEP20/31.2 | *Acinetobacter pitti* |
| SM/U/SEP20/31 | SM/U/SEP20/31.3 | *Klebsiella oxytoca* |
| SM/U/SEP20/31 | SM/U/SEP20/31.4 | *Enterobacter cloacae* |
| SM/U/SEP20/32 | SM/U/SEP20/32.1 | Unidentified |
| SM/U/SEP20/32 | SM/U/SEP20/32.2 | *Acinetobacter pitti* |
| SM/U/SEP20/32 | SM/U/SEP20/32.3 | *Klebsiella oxytoca* |
| SM/U/SEP20/32 | SM/U/SEP20/32.4 | *Pseudomonas putida* |
| SM/U/SEP20/33 | SM/U/SEP20/33.1 | Unidentified |
| SM/U/SEP20/33 | SM/U/SEP20/33.2 | *Cupriavidus necator* |
| SM/U/SEP20/34 | SM/U/SEP20/34.1 | *Enterobacter hormaechei* |
| SM/U/SEP20/34 | SM/U/SEP20/34.2 | *Cupriavidus necator* |
| SM/U/SEP20/35 | SM/U/SEP20/35.1 | Unidentified |
| SM/U/SEP20/35 | SM/U/SEP20/35.2 | *Serratia marcescens* |
| SM/U/SEP20/35 | SM/U/SEP20/35.3 | *Pseudomonas putida* |
| SM/U/SEP20/36 | SM/U/SEP20/36.1 | *Klebsiella oxytoca* |
| SM/U/SEP20/36 | SM/U/SEP20/36.2 | *Pseudomonas putida* |
| SM/U/SEP20/36 | SM/U/SEP20/36.3 | *Escherichia coli* |
| SM/U/SEP20/37 | SM/U/SEP20/37.1 | *Citrobacter braaki* |
| SM/U/SEP20/37 | SM/U/SEP20/37.2 | *Klebsiella pneumoniae* |
| SM/U/SEP20/37 | SM/U/SEP20/37.3 | *Klebsiella aerogenes* |
| SM/U/SEP20/37 | SM/U/SEP20/37.4 | *Pseudomonas mosselii* |
| SM/U/SEP20/38 | SM/U/SEP20/38.1 | *Escherichia coli* |
| SM/U/SEP20/38 | SM/U/SEP20/38.2 | *Acinetobacter gyllenbergii* |
| SM/U/SEP20/38 | SM/U/SEP20/38.3 | *Enterobacter cloacae* |
| SM/U/SEP20/38 | SM/U/SEP20/38.4 | Unidentified |
| SM/U/SEP20/39 | SM/U/SEP20/39.1 | *Leclercia adecarboxylata* |
| SM/U/SEP20/39 | SM/U/SEP20/39.2 | *Cupriavidus necator* |
| SM/U/SEP20/39 | SM/U/SEP20/39.3 | *Pseudomonas putida* |
| SM/U/SEP20/39 | SM/U/SEP20/39.4 | *Pseudomonas aeruginosa* |
| SM/U/SEP20/39 | SM/U/SEP20/39.5 | *Cupriavidus necator* |
| SM/U/SEP20/40 | SM/U/SEP20/40.1 | *Escherichia coli* |
| SM/U/SEP20/40 | SM/U/SEP20/40.2 | Unidentified |
| SM/U/SEP20/40 | SM/U/SEP20/40.3 | *Enterobacter cloacae* |
| SM/U/SEP20/40 | SM/U/SEP20/40.4 | Unidentified |
| SM/H/DEC20/01 | SM/H/DEC20/1.1 | *Pseudomonas mosselii* |
| SM/H/DEC20/01 | SM/H/DEC20/1.2 | *Cupriavidus necator* |
| SM/H/DEC20/02 | SM/H/DEC20/2.1 | *Stenotrophomonas maltophilia* |
| SM/H/DEC20/02 | SM/H/DEC20/2.2 | *Rhizobium radiobacter* |
| SM/H/DEC20/02 | SM/H/DEC20/2.3 | *Pantoea ananatis* |
| SM/H/DEC20/02 | SM/H/DEC20/2.4 | *Enterobacter cloacae / asburiae* |
| SM/H/DEC20/03 | SM/H/DEC20/3.1 | *Pseudomonas putida* |
| SM/H/DEC20/03 | SM/H/DEC20/3.2 | *Enterobacter cloacae / asburiae* |
| SM/H/DEC20/03 | SM/H/DEC20/3.3 | *Stenotrophomonas maltophilia* |
| SM/H/DEC20/03 | SM/H/DEC20/3.4 | *Enterobacter hormacheii* |
| SM/H/DEC20/04 | SM/H/DEC20/4.1 | *Enterobacter cloacae / asburiae* |
| SM/H/DEC20/04 | SM/H/DEC20/4.2 | Unidentified |
| SM/H/DEC20/04 | SM/H/DEC20/4.3 | *Pantoea agglomerans* |
| SM/H/DEC20/05 | SM/H/DEC20/5.1 | *Acinetobacter calcoaceticus* |
| SM/H/DEC20/05 | SM/H/DEC20/5.2 | *Enterobacter cloacae / asburiae* |
| SM/H/DEC20/05 | SM/H/DEC20/5.3 | *Rhizobium radiobacter* |
| SM/H/DEC20/06 | SM/H/DEC20/6.1 | *Acinetobacter calcoaceticus* |
| SM/H/DEC20/06 | SM/H/DEC20/6.2 | Unidentified |
| SM/H/DEC20/06 | SM/H/DEC20/6.3 | Unidentified |
| SM/H/DEC20/07 | SM/H/DEC20/7.1 | *Pseudescherichia valneris* |
| SM/H/DEC20/07 | SM/H/DEC20/7.2 | *Cupriavidus necator* |
| SM/H/DEC20/07 | SM/H/DEC20/7.3 | *Leclercia adecarboxylata* |
| SM/H/DEC20/07 | SM/H/DEC20/7.4 | *Rhizobium radiobacter* |
| SM/H/DEC20/08 | SM/H/DEC20/8.1 | *Pantoea dispersa* |
| SM/H/DEC20/08 | SM/H/DEC20/8.2 | Unidentified |
| SM/H/DEC20/08 | SM/H/DEC20/8.3 | *Pantoea agglomerans* |
| SM/H/DEC20/09 | SM/H/DEC20/9.1 | Unidentified |
| SM/H/DEC20/09 | SM/H/DEC20/9.2 | *Pseudomonas putida* |
| SM/H/DEC20/09 | SM/H/DEC20/9.3 | *Rhizobium radiobacter* |
| SM/H/DEC20/09 | SM/H/DEC20/9.4 | Unidentified |
| SM/H/DEC20/09 | SM/H/DEC20/9.5 | Unidentified |
| SM/H/DEC20/09 | SM/H/DEC20/9.6 | *Achromobacter xylosoxidans/denitrificans* |
| SM/H/DEC20/09 | SM/H/DEC20/9.7 | *Enterobacter kobei* |
| SM/H/DEC20/10 | SM/H/DEC20/10.1 | *Pseudomonas mosselii* |
| SM/H/DEC20/10 | SM/H/DEC20/10.2 | *Leclercia adecarboxylata* |
| SM/H/DEC20/10 | SM/H/DEC20/10.3 | *Escherichia harmannii* |
| SM/H/DEC20/10 | SM/H/DEC20/10.4 | *Rhizobium radiobacter* |
| SM/H/DEC20/11 | SM/H/DEC20/11.1 | Unidentified |
| SM/H/DEC20/11 | SM/H/DEC20/11.2 | *Pseudomonas putida* |
| SM/H/DEC20/11 | SM/H/DEC20/11.3 | Unidentified |
| SM/H/DEC20/12 | SM/H/DEC20/12.1 | *Pseudomonas putida* |
| SM/H/DEC20/12 | SM/H/DEC20/12.2 | Unidentified |
| SM/H/DEC20/13 | SM/H/DEC20/13.1 | *Pseudomonas mosselii* |
| SM/H/DEC20/13 | SM/H/DEC20/13.2 | Unidentified |
| SM/H/DEC20/14 | SM/H/DEC20/14.1 | *Pseudomonas putida* |
| SM/H/DEC20/15 | SM/H/DEC20/15.1 | Unidentified |
| SM/H/DEC20/15 | SM/H/DEC20/15.2 | *Pseudomonas putida* |
| SM/H/DEC20/15 | SM/H/DEC20/15.3 | *Ochrobactrum intermedium* |
| SM/H/DEC20/16 | SM/H/DEC20/16.1 | Unidentified |
| SM/H/DEC20/16 | SM/H/DEC20/16.2 | *Pantoea dispersa* |
| SM/H/DEC20/16 | SM/H/DEC20/16.3 | Unidentified |
| SM/H/DEC20/16 | SM/H/DEC20/16.4 | *Enterobacter hormacheii* |
| SM/H/DEC20/17 | SM/H/DEC20/17.1 | *Enterobacter hormacheii* |
| SM/H/DEC20/17 | SM/H/DEC20/17.2 | Unidentified |
| SM/H/DEC20/17 | SM/H/DEC20/17.3 | *Cupriavidus necator* |
| SM/H/DEC20/17 | SM/H/DEC20/17.4 | *Pseudomonas species (mosselii / putida)* |
| SM/H/DEC20/18 | SM/H/DEC20/18.1 | *Cupriavidus necator* |
| SM/H/DEC20/18 | SM/H/DEC20/18.2 | *Stenotrophomonas maltophilia* |
| SM/H/DEC20/19 | SM/H/DEC20/19.1 | *Pantoea agglumerans* |
| SM/H/DEC20/19 | SM/H/DEC20/19.2 | *Pseudomonas putida* |
| SM/H/DEC20/20 | SM/H/DEC20/20.1 | *Enterobacter hormacheii* |
| SM/H/DEC20/20 | SM/H/DEC20/20.2 | *Enterobacter hormacheii* |
| SM/H/DEC20/20 | SM/H/DEC20/20.3 | Unidentified |
| SM/H/DEC20/20 | SM/H/DEC20/20.4 | *Klebsiella pnneumoniae* |
| SM/U/DEC20/21 | SM/U/DEC20/21.1 | No growth |
| SM/U/DEC20/21 | SM/U/DEC20/21.2 | *Pseudomonas mendocina* |
| SM/U/DEC20/21 | SM/U/DEC20/21.3 | *Acinetobacter pitti* |
| SM/U/DEC20/22 | SM/U/DEC20/22.1 | Unidentified |
| SM/U/DEC20/22 | SM/U/DEC20/22.2 | *Pseudomonas putida* |
| SM/U/DEC20/22 | SM/U/DEC20/22.3 | *Pseudomonas putida* |
| SM/U/DEC20/22 | SM/U/DEC20/22.4 | *Citrobacter amalonaticus* |
| SM/U/DEC20/22 | SM/U/DEC20/22.5 | Unidentified |
| SM/U/DEC20/22 | SM/U/DEC20/22.6 | *Acinetobacter lwoffii* |
| SM/U/DEC20/23 | SM/U/DEC20/23.1 | *Enterobacter cloacae* |
| SM/U/DEC20/23 | SM/U/DEC20/23.2 | *Pseudomonas mendocina* |
| SM/U/DEC20/23 | SM/U/DEC20/23.3 | *Cupriavidus necator* |
| SM/U/DEC20/23 | SM/U/DEC20/23.4 | *Rhizobium radiobacter* |
| SM/U/DEC20/24 | SM/U/DEC20/24.1 | No growth |
| SM/U/DEC20/24 | SM/U/DEC20/24.2 | *Bacillus sporothermodurans* |
| SM/U/DEC20/24 | SM/U/DEC20/24.3 | No growth |
| SM/U/DEC20/25 | SM/U/DEC20/25.1 | No growth |
| SM/U/DEC20/25 | SM/U/DEC20/25.2 | *Pseudomonas putida* |
| SM/U/DEC20/25 | SM/U/DEC20/25.3 | *Rhizobium radiobacter* |
| SM/U/DEC20/26 | SM/U/DEC20/26.1 | *Pseudomonas aeruginosa* |
| SM/U/DEC20/26 | SM/U/DEC20/26.2 | *Pseudomonas aeruginosa* |
| SM/U/DEC20/26 | SM/U/DEC20/26.3 | *Citrobacter freundii* |
| SM/U/DEC20/26 | SM/U/DEC20/26.4 | *Staphylococcus sciuri* |
| SM/U/DEC20/27 | SM/U/DEC20/27.1 | *Pseudomonas aeruginosa* |
| SM/U/DEC20/27 | SM/U/DEC20/27.2 | *Pseudomonas putida* |
| SM/U/DEC20/27 | SM/U/DEC20/27.3 | Unidentified |
| SM/U/DEC20/28 | SM/U/DEC20/28.1 | *Escherichia coli* |
| SM/U/DEC20/28 | SM/U/DEC20/28.2 | Unidentified |
| SM/U/DEC20/28 | SM/U/DEC20/28.3 | Unidentified |
| SM/U/DEC20/28 | SM/U/DEC20/28.4 | *Pseudomonas aeruginosa* |
| SM/U/DEC20/28 | SM/U/DEC20/28.5 | *Acinetobacter pitti* |
| SM/U/DEC20/28 | SM/U/DEC20/28.6 | *Enterobacter cloacae* |
| SM/U/DEC20/29 | SM/U/DEC20/29.1 | *Pseudomonas putida* |
| SM/U/DEC20/29 | SM/U/DEC20/29.2 | *Acinetobacter pitti* |
| SM/U/DEC20/29 | SM/U/DEC20/29.3 | *Serratia marscesens* |
| SM/U/DEC20/29 | SM/U/DEC20/29.4 | Unidentified |
| SM/U/DEC20/29 | SM/U/DEC20/29.5 | *Acinetobacter pitti* |
| SM/U/DEC20/30 | SM/U/DEC20/30.1 | Unidentified |
| SM/U/DEC20/30 | SM/U/DEC20/30.2 | Unidentified |
| SM/U/DEC20/30 | SM/U/DEC20/30.3 | Unidentified |
| SM/U/DEC20/30 | SM/U/DEC20/30.4 | Unidentified |
| SM/U/DEC20/31 | SM/U/DEC20/31.1 | Unidentified |
| SM/U/DEC20/31 | SM/U/DEC20/31.2 | *Pseudomonas mendocina* |
| SM/U/DEC20/31 | SM/U/DEC20/31.3 | *Pantoea agglumerans* |
| SM/U/DEC20/32 | SM/U/DEC20/32.1 | *Acinetobacter pitti* |
| SM/U/DEC20/32 | SM/U/DEC20/32.2 | *Citrobacter amalonaticus* |
| SM/U/DEC20/33 | SM/U/DEC20/33.1 | *Pseudomonas putida* |
| SM/U/DEC20/33 | SM/U/DEC20/33.2 | *Pseudomonas putida* |
| SM/U/DEC20/34 | SM/U/DEC20/34.1 | Unidentified |
| SM/U/DEC20/34 | SM/U/DEC20/34.2 | *Pseudomonas alcaligenes* |
| SM/U/DEC20/34 | SM/U/DEC20/34.3 | *Cupriavidus necator* |
| SM/U/DEC20/34 | SM/U/DEC20/34.4 | *Pseudomonas mendocina* |
| SM/U/DEC20/35 | SM/U/DEC20/35.1 | *Enterobacter asburiae* |
| SM/U/DEC20/35 | SM/U/DEC20/35.2 | *Acinetobacter pitti* |
| SM/U/DEC20/35 | SM/U/DEC20/35.3 | *Siccibacter turicensis* |
| SM/U/DEC20/36 | SM/U/DEC20/36.1 | *Cupriavidus necator* |
| SM/U/DEC20/36 | SM/U/DEC20/36.2 | Unidentified |
| SM/U/DEC20/37 | SM/U/DEC20/37.1 | *Pseudomonas mosellii* |
| SM/U/DEC20/37 | SM/U/DEC20/37.2 | *Cupriavidus necator* |
| SM/U/DEC20/38 | SM/U/DEC20/38.1 | *Escherichia coli* |
| SM/U/DEC20/38 | SM/U/DEC20/38.2 | *Pseudoescherichia vulneris* |
| SM/U/DEC20/38 | SM/U/DEC20/38.3 | *Staphylococcus scuiri* |
| SM/U/DEC20/39 | SM/U/DEC20/39.1 | *Enterobacter homaechei* |
| SM/U/DEC20/39 | SM/U/DEC20/39.2 | *Klebsiella pneumoniae* |
| SM/U/DEC20/39 | SM/U/DEC20/39.3 | Unidentified |
| SM/U/DEC20/39 | SM/U/DEC20/39.4 | *Enterobacter cancerogenes* |
| SM/U/DEC20/40 | SM/U/DEC20/40.1 | *Enterobacter asburiae / cloacae* |
| SM/U/DEC20/40 | SM/U/DEC20/40.2 | *Enterobacter homaechei* |
| SM/U/DEC20/40 | SM/U/DEC20/40.3 | *Enterobacter asburiae / cloacae* |
| SM/U/DEC20/40 | SM/U/DEC20/40.4 | *Acinetobacter calcoaceticus* |
| SM/H/APR21/01 | SM/H/APR21/1.1 | *Aeromonas hydrophila* |
| SM/H/APR21/01 | SM/H/APR21/1.2 | *Pseudomonas mendocina* |
| SM/H/APR21/02 | SM/H/APR21/2.1 | *Pseudomonas mosellii* |
| SM/H/APR21/02 | SM/H/APR21/2.2 | *Acinetobacter pitti* |
| SM/H/APR21/02 | SM/H/APR21/2.3 | *Pantoea ananatis* |
| SM/H/APR21/03 | SM/H/APR21/3.1 | *Pseudomonas alcaligenes* |
| SM/H/APR21/03 | SM/H/APR21/3.2 | *Franconibacter pulveris* |
| SM/H/APR21/03 | SM/H/APR21/3.3 | Unidentified |
| SM/H/APR21/04 | SM/H/APR21/4.1 | Unidentified |
| SM/H/APR21/04 | SM/H/APR21/4.2 | Unidentified |
| SM/H/APR21/05 | SM/H/APR21/5.1 | *Rhizobium radiobacter* |
| SM/H/APR21/05 | SM/H/APR21/5.2 | Unidentified |
| SM/H/APR21/06 | SM/H/APR21/6.1 | Unidentified |
| SM/H/APR21/06 | SM/H/APR21/6.2 | Unidentified |
| SM/H/APR21/07 | SM/H/APR21/7.1 | Unidentified |
| SM/H/APR21/07 | SM/H/APR21/7.2 | *Pseudomonas mendocina* |
| SM/H/APR21/08 | SM/H/APR21/8.1 | *Acinetobacter junii* |
| SM/H/APR21/08 | SM/H/APR21/8.2 | *Pseudomonas putida* |
| SM/H/APR21/09 | SM/H/APR21/9.1 | *Rhizobium radiobacter* |
| SM/H/APR21/09 | SM/H/APR21/9.2 | Unidentified |
| SM/H/APR21/10 | SM/H/APR21/10.1 | *Enterobacter cloacae / asburiae* |
| SM/H/APR21/10 | SM/H/APR21/10.2 | *Pseudomonas putida* |
| SM/H/APR21/11 | SM/H/APR21/11.1 | Unidentified |
| SM/H/APR21/11 | SM/H/APR21/11.2 | *Rhizobium radiobacter* |
| SM/H/APR21/12 | SM/H/APR21/12.1 | *Pseudomonas aeruginosa* |
| SM/H/APR21/12 | SM/H/APR21/12.2 | *Acinetobacter pitti* |
| SM/H/APR21/13 | SM/H/APR21/13.1 | Unidentified |
| SM/H/APR21/13 | SM/H/APR21/13.2 | *Pseudomonas stutzeri* |
| SM/H/APR21/14 | SM/H/APR21/14.1 | *Burholderia vietnamiensis* |
| SM/H/APR21/14 | SM/H/APR21/14.2 | Unidentified |
| SM/H/APR21/15 | SM/H/APR21/15.1 | *Rhizobium radiobacter* |
| SM/H/APR21/15 | SM/H/APR21/15.2 | Unidentified |
| SM/H/APR21/15 | SM/H/APR21/15.3 | *Pantoea dispersa* |
| SM/H/APR21/15 | SM/H/APR21/15.4 | Unidentified |
| SM/H/APR21/16 | SM/H/APR21/16.1 | Unidentified |
| SM/H/APR21/16 | SM/H/APR21/16.2 | Unidentified |
| SM/H/APR21/16 | SM/H/APR21/16.3 | Unidentified |
| SM/H/APR21/17 | SM/H/APR21/17.1 | Unidentified |
| SM/H/APR21/17 | SM/H/APR21/17.2 | Unidentified |
| SM/H/APR21/17 | SM/H/APR21/17.3 | *Rhizobium radiobacter* |
| SM/H/APR21/18 | SM/H/APR21/18.1 | *Burholderia vietnamiensis* |
| SM/H/APR21/18 | SM/H/APR21/18.2 | Unidentified |
| SM/H/APR21/19 | SM/H/APR21/19.1 | Unidentified |
| SM/H/APR21/19 | SM/H/APR21/19.2 | *Pantoea dispersa* |
| SM/H/APR21/19 | SM/H/APR21/19.3 | Unidentified |
| SM/H/APR21/19 | SM/H/APR21/19.4 | Unidentified |
| SM/H/APR21/20 | SM/H/APR21/20.1 | *Acinetobacter radioresistans* |
| SM/H/APR21/20 | SM/H/APR21/20.2 | *Pantoea agglomerans* |
| SM/H/APR21/20 | SM/H/APR21/20.3 | Unidentified |
| SM/H/APR21/20 | SM/H/APR21/20.4 | Unidentified |
| SM/H/APR21/21 | SM/H/APR21/21.1 | Unidentified |
| SM/H/APR21/21 | SM/H/APR21/21.2 | Unidentified |
| SM/H/APR21/21 | SM/H/APR21/21.3 | Unidentified |
| SM/H/APR21/22 | SM/H/APR21/22.1 | Unidentified |
| SM/H/APR21/22 | SM/H/APR21/22.2 | *Cupriavidus necator* |
| SM/H/APR21/22 | SM/H/APR21/22.3 | *Pseudomonas aeruginosa* |
| SM/H/APR21/23 | SM/H/APR21/23.1 | *Enterobacter cloacae/asburiae* |
| SM/H/APR21/23 | SM/H/APR21/23.2 | *Stenotrophomonas maltophilia* |
| SM/H/APR21/24 | SM/H/APR21/24.1 | *Pseudomonas alcaligenes* |
| SM/H/APR21/24 | SM/H/APR21/24.2 | Unidentified |
| SM/H/APR21/25 | SM/H/APR21/25.1 | *Pseudomonas putida* |
| SM/H/APR21/25 | SM/H/APR21/25.2 | *Enterobacter cloacae* |
| SM/H/APR21/26 | SM/H/APR21/26.1 | *Citrobacter braaki/freundii* |
| SM/H/APR21/26 | SM/H/APR21/26.2 | *Enterobacter hormaechei* |
| SM/H/APR21/26 | SM/H/APR21/26.3 | *Pseudomonas alcaligenes* |
| SM/H/APR21/27 | SM/H/APR21/27.1 | Unidentified |
| SM/H/APR21/27 | SM/H/APR21/27.2 | *Pseudomonas putida* |
| SM/H/APR21/28 | SM/H/APR21/28.1 | Unidentified |
| SM/H/APR21/28 | SM/H/APR21/28.2 | Unidentified |
| SM/H/APR21/28 | SM/H/APR21/28.3 | Unidentified |
| SM/H/APR21/29 | SM/H/APR21/29.1 | *Pseudomonas putida* |
| SM/H/APR21/29 | SM/H/APR21/29.2 | *Enterobacter cloacae/asburiae* |
| SM/H/APR21/29 | SM/H/APR21/29.3 | *Serratia marcescens* |
| SM/H/APR21/30 | SM/H/APR21/30.1 | Unidentified |
| SM/H/APR21/30 | SM/H/APR21/30.2 | *Leclaria decarboxylata* |
| SM/H/APR21/30 | SM/H/APR21/30.3 | Unidentified |
| SM/H/APR21/30 | SM/H/APR21/30.4 | *Escherichia coli* |
| SM/H/APR21/31 | SM/H/APR21/31.1 | Unidentified |
| SM/H/APR21/31 | SM/H/APR21/31.2 | Unidentified |
| SM/H/APR21/32 | SM/H/APR21/32.1 | Unidentified |
| SM/H/APR21/32 | SM/H/APR21/32.2 | Unidentified |
| SM/H/APR21/33 | SM/H/APR21/33.1 | Unidentified |
| SM/H/APR21/33 | SM/H/APR21/33.2 | *Rhizobium radiobacter* |
| SM/H/APR21/34 | SM/H/APR21/34.1 | Unidentified |
| SM/H/APR21/34 | SM/H/APR21/34.2 | *Enterobacter cloacae/asburiae* |
| SM/H/APR21/34 | SM/H/APR21/34.3 | Unidentified |
| SM/H/APR21/34 | SM/H/APR21/34.4 | Unidentified |
| SM/H/APR21/35 | SM/H/APR21/35.1 | *Rhizobium radiobacter* |
| SM/H/APR21/35 | SM/H/APR21/35.2 | *Pseudomonas alcaligenes* |
| SM/H/APR21/36 | SM/H/APR21/36.1 | *Siccibacter turicensis/Cronobacter malonaticus* |
| SM/H/APR21/36 | SM/H/APR21/36.2 | *Acinetobacter pitti* |
| SM/H/APR21/36 | SM/H/APR21/36.3 | Unidentified |
| SM/H/APR21/37 | SM/H/APR21/37.1 | *Pseudomonas straminea* |
| SM/H/APR21/37 | SM/H/APR21/37.2 | *Pseudomonas straminea* |
| SM/H/APR21/37 | SM/H/APR21/37.3 | *Pantoea dispersa* |
| SM/H/APR21/38 | SM/H/APR21/38.1 | Unidentified |
| SM/H/APR21/38 | SM/H/APR21/38.2 | *Acinetobacter radioresistans* |
| SM/H/APR21/38 | SM/H/APR21/38.3 | *Acinetobacter radioresistans* |
| SM/H/APR21/39 | SM/H/APR21/39.1 | *Cupriavidus necator* |
| SM/H/APR21/39 | SM/H/APR21/39.2 | *Siccibacter turicensis* |
| SM/H/APR21/39 | SM/H/APR21/39.3 | *Pseudomonas putida* |
| SM/H/APR21/40 | SM/H/APR21/40.1 | Unidentified |
| SM/H/APR21/40 | SM/H/APR21/40.2 | *Acinetobacter pitti* |
| SM/H/APR21/40 | SM/H/APR21/40.3 | Unidentified |

Organisms isolated by culture from different sampling spots (1-40) and the isolates sub-numbered based on the sampling spot (H- hospital, U- university*)*.

Supplementary Table 2

**Table 2:** **AST pattern of the Pseudomonas and Acinetobacter isolates obtained from from each of the soil samples from JSS Hospital and JSS University**.

| **Sample ID** | **ORGANISM** | **TIC** | **PIT** | **CAZ** | **SCF** | **CEF** | **DOR** | **IMP** | **MRP** | **AK** | **GEN** | **CIP** | **LEV** | **MIN** | **TGC** | **CL** | **COT** |
| --- | --- | --- | --- | --- | --- | --- | --- | --- | --- | --- | --- | --- | --- | --- | --- | --- | --- |
| SM/H/SEP20/1.1 | *Pseudomonas putida* | R | S | S | S | S | - | S | S | S | S | S | S | - | R | - | R |
| SM/H/SEP20/2.2 | *Pseudomonas mosselii* | R | S | S | S | S | - | S | I | S | S | S | S | - | R | - | R |
| SM/H/SEP20/2.3 | *Pseudomonas putida* | R | S | S | S | S | - | S | S | S | S | S | S | - | R | - | R |
| SM/H/SEP20/3.3 | *Pseudomonas putida* | R | S | S | S | S | - | S | S | S | S | S | S | - | R | - | R |
| SM/H/SEP20/4.4 | *Pseudomonas putida* | R | S | S | S | S | - | S | S | S | S | S | S | - | R | - | R |
| SM/H/SEP20/5.2 | *Pseudomonas putida* | R | S | S | S | S | - | S | S | S | S | S | S | - | R | - | R |
| SM/H/AUG20/7.2 | *Pseudomonas alcaligenes* | S | S | S | S | S | - | S | S | S | S | S | S | - | R | - | S |
| SM/H/AUG20/8.1 | *Pseudomonas putida* | R | S | S | S | S | - | S | S | S | S | S | S | - | R | - | R |
| SM/H/SEP20/10.2 | *Pseudomonas mosselii* | R | S | S | S | S | - | S | S | S | S | S | S | - | R | - | R |
| SM/H/SEP20/11.2 | *Pseudomonas mosselii* | R | S | S | S | S | - | S | S | S | S | S | S | - | R | - | R |
| SM/H/SEP20/13.3 | *Pseudomonas putida* | R | S | S | S | S | - | S | S | S | S | S | S | - | R | - | R |
| SM/H/SEP20/13.4 | *Pseudomonas putida* | R | S | S | S | S | - | S | S | S | S | S | S | - | R | - | R |
| SM/H/SEP20/14.2 | *Pseudomonas mosselii* | R | S | S | S | S | - | S | S | S | S | S | S | - | R | - | S |
| SM/H/AUG20/17.1 | *Pseudomonas putida* | R | S | S | S | S | - | S | S | S | S | S | S | - | R | - | R |
| SM/H/AUG20/18.2 | *Pseudomonas putida* | R | I | I | R | R | - | S | S | S | S | S | S | - | R | - | R |
| SM/H/SEP20/20.1 | *Pseudomonas putida* | R | S | S | S | S | - | S | S | S | S | S | S | - | R | - | R |
| SM/U/SEP20/22.1 | *Pseudomonas putida* | R | S | S | S | S | - | S | S | S | S | S | S | - | R | - | R |
| SM/U/SEP20/23.1 | *Pseudomonas putida* | R | S | S | S | S | - | S | S | S | S | S | S | - | R | - | R |
| SM/U/SEP20/25.2 | *Pseudomonas putida* | R | S | S | S | S | - | S | S | S | S | S | S | - | R | - | R |
| SM/U/SEP20/26.2 | *Pseudomonas putida* | R | S | S | S | S | - | S | S | S | S | S | S | - | R | - | R |
| SM/U/SEP20/28.1 | *Pseudomonas putida* | R | S | S | S | S | - | S | S | S | S | S | S | - | R | - | R |
| SM/U/SEP20/31.1 | *Pseudomonas putida* | R | S | S | S | S | - | S | S | S | S | S | S | - | R | - | R |
| SM/U/SEP20/32.4 | *Pseudomonas putida* | R | S | S | S | S | - | S | S | S | S | S | S | - | R | - | R |
| SM/U/SEP20/35.3 | *Pseudomonas putida* | R | S | S | S | S | - | S | S | S | S | S | S | - | R | - | R |
| SM/U/SEP20/36.2 | *Pseudomonas putida* | R | S | S | S | S | - | S | S | S | S | S | S | - | R | - | R |
| SM/U/SEP20/37.4 | *Pseudomonas mosselii* | R | S | S | S | S | - | R | S | S | S | S | S | - | R | - | R |
| SM/U/SEP20/39.4 | *Pseudomonas aeruginosa* | R | S | S | S | S | - | S | S | S | S | S | S | - | R | - | - |
| SM/H/DEC20/1.1 | *Pseudomonas mosselii* | R | S | S | S | S | - | S | S | S | S | S | S | - | R | - | R |
| SM/H/DEC20/3.1 | *Pseudomonas putida* | R | S | S | S | S | - | S | S | S | S | S | S | - | R | - | R |
| SM/H/DEC20/9.2 | *Pseudomonas putida* | R | S | S | S | S | - | S | S | S | S | S | S | - | R | - | R |
| SM/H/DEC20/10.1 | *Pseudomonas mosselii* | R | S | S | S | S | - | R | S | S | S | S | S | - | R | - | R |
| SM/H/DEC20/11.2 | *Pseudomonas putida* | R | S | S | S | S | - | S | S | S | S | S | S | - | R | - | R |
| SM/H/DEC20/12.1 | *Pseudomonas putida* | R | S | S | S | S | - | S | S | S | S | S | S | - | R | - | R |
| SM/H/DEC20/13.1 | *Pseudomonas mosselii* | R | S | S | S | S | - | S | S | S | S | S | S | - | R | - | R |
| SM/H/DEC20/14.1 | *Pseudomonas putida* | R | S | S | S | S | - | S | S | S | S | S | S | - | R | - | R |
| SM/H/DEC20/15.2 | *Pseudomonas putida* | R | S | S | S | S | - | S | S | S | S | S | S | - | R | - | R |
| SM/H/DEC20/17.4 | *Pseudomonas species* | R | S | S | S | S | - | S | S | S | S | S | S | - | R | - | R |
| SM/H/DEC20/19.2 | *Pseudomonas putida* | R | S | S | S | S | - | S | S | S | S | S | S | - | R | - | R |
| SM/U/DEC20/21.2 | *Pseudomonas mendocina* | S | S | S | S | S | - | S | S | S | S | S | S | - | R | - | S |
| SM/U/DEC20/22.2 | *Pseudomonas putida* | R | S | S | S | S | - | S | S | S | S | S | S | - | R | - | R |
| SM/U/DEC20/22.3 | *Pseudomonas putida* | S | S | S | S | S | - | S | S | S | S | S | S | - | R | - | S |
| SM/U/DEC20/23.2 | *Pseudomonas mendocina* | S | S | S | S | S | - | S | S | S | S | S | S | - | R | - | S |
| SM/U/DEC20/25.2 | *Pseudomonas putida* | R | S | S | S | S | - | S | S | S | S | S | S | - | R | - | S |
| SM/U/DEC20/26.1 | *Pseudomonas aeruginosa* | S | S | S | S | S | - | S | S | S | S | S | S | - | R | - | - |
| SM/U/DEC20/26.2 | *Pseudomonas aeruginosa* | S | S | S | S | S | - | S | S | S | S | S | S | - | R | - | - |
| SM/U/DEC20/27.1 | *Pseudomonas aeruginosa* | S | S | S | S | S | - | S | S | S | S | S | S | - | R | - | - |
| SM/U/DEC20/27.2 | *Pseudomonas putida* | R | R | R | R | R | - | S | S | S | S | S | S | - | R | - | R |
| SM/U/DEC20/28.4 | *Pseudomonas aeruginosa* | S | S | S | S | S | - | S | S | S | S | S | S | - | R | - | - |
| SM/U/DEC20/29.1 | *Pseudomonas putida* | R | S | S | S | S | - | S | S | S | S | S | S | - | R | - | R |
| SM/U/DEC20/31.2 | *Pseudomonas mendocina* | S | S | S | S | S | - | S | S | S | S | S | S | - | R | - | S |
| SM/U/DEC20/33.1 | *Pseudomonas putida* | R | S | S | S | S | - | S | S | S | S | S | S | - | R | - | R |
| SM/U/DEC20/33.2 | *Pseudomonas putida* | R | S | S | S | S | - | S | S | S | S | S | S | - | R | - | R |
| SM/U/DEC20/34.2 | *Pseudomonas aeruginosa* | S | S | S | S | S | - | S | S | S | S | S | S | - | R | - | - |
| SM/U/DEC20/34.4 | *Pseudomonas mendocina* | S | S | S | S | S | - | S | S | S | S | S | S | - | R | - | S |
| SM/U/DEC20/37.1 | *Pseudomonas mosselii* | R | S | S | S | S | - | S | S | S | S | S | S | - | R | - | S |
| SM/H/APR21/1.2 | *Pseudomonas mendocina* | S | S | S | S | S | - | S | S | S | S | S | S | - | R | - | S |
| SM/H/APR21/2.1 | *Pseudomonas mosselii* | R | S | S | S | S | - | S | S | S | S | S | S | - | R | - | S |
| SM/H/APR21/3.1 | *Pseudomonas alcaligenes* | S | S | S | S | S | - | S | S | S | S | S | S | - | R | - | R |
| SM/H/APR21/7.2 | *Pseudomonas mendocina* | S | S | S | S | S | - | S | S | S | S | S | S | - | R | - | S |
| SM/H/APR21/8.2 | *Pseudomonas putida* | R | S | S | S | S | - | S | S | S | S | S | S | - | R | - | R |
| SM/H/APR21/10.2 | *Pseudomonas putida* | R | S | S | S | S | - | S | S | S | S | S | S | - | R | - | R |
| SM/H/APR21/12.1 | *Pseudomonas aeruginosa* | S | S | S | S | S | - | S | S | S | S | S | S | - | R | - | - |
| SM/H/APR21/13.2 | *Pseudomonas stutzeri* | S | S | S | S | S | - | S | S | S | S | S | S | - | R | - | S |
| SM/U/APR21/22.3 | *Pseudomonas aeruginosa* | S | S | S | S | S | - | S | S | S | S | S | S | - | R | - | - |
| SM/U/APR21/24.1 | *Pseudomonas alcaligenes* | S | S | S | S | S | - | S | S | S | S | S | S | - | R | - | S |
| SM/U/APR21/25.1 | *Pseudomonas putida* | R | S | S | S | S | - | S | S | S | S | S | S | - | R | - | R |
| SM/U/APR21/26.3 | *Pseudomonas alcaligenes* | S | S | S | S | S | - | S | S | S | S | S | S | - | R | - | S |
| SM/U/APR21/27.2 | *Pseudomonas putida* | R | S | S | S | S | - | S | S | S | S | S | S | - | R | - | R |
| SM/U/APR21/29.1 | *Pseudomonas putida* | R | S | S | S | S | - | S | S | S | S | S | S | - | R | - | R |
| SM/U/APR21/35.2 | *Pseudomonas alcaligenes* | S | S | S | S | S | - | S | S | S | S | S | S | - | R | - | S |
| SM/U/APR21/37.1 | *Pseudomonas straminea* | S | S | S | S | S | - | S | S | S | R | S | S | - | R | - | S |
| SM/U/APR21/37.2 | *Pseudomonas straminea* | S | S | S | S | S | - | S | S | S | R | S | S | - | R | - | S |
| SM/U/APR21/39.3 | *Pseudomonas putida* | R | S | S | S | S | - | S | S | S | S | S | S | - | R | - | R |
| SM/H/SEP20/5.1 | *Acinetobacter pitti* | S | S | S | S | S | S | S | S | - | S | S | S | S | S | I | S |
| SM/H/SEP20/10.3 | *Acinetobacter pitti* | S | S | S | S | S | S | S | S | - | S | S | S | S | S | I | S |
| SM/H/SEP20/11.4 | *Acinetobacter pitti* | S | S | S | S | S | S | S | S | - | S | S | S | S | S | I | S |
| SM/H/SEP20/12.3 | *Acinetobacter pitti* | S | S | S | S | S | S | S | S | - | S | S | S | S | S | I | S |
| SM/H/SEP20/15.2 | *Acinetobacter pitti* | S | I | S | S | S | S | S | S | - | S | S | S | S | S | I | S |
| SM/H/AUG20/17.3 | *Acinetobacter pitti* | S | S | S | S | S | S | S | S | - | S | S | S | S | S | I | S |
| SM/H/SEP20/19.3 | *Acinetobacter pitti* | S | S | S | S | S | S | S | S | - | S | S | S | S | S | I | S |
| SM/H/SEP20/20.3 | *Acinetobacter pitti* | S | S | S | S | S | S | S | S | - | S | S | S | S | S | I | S |
| SM/U/SEP20/21.3 | *Acinetobacter pitti* | S | I | S | S | S | S | S | S | - | S | S | S | S | S | I | S |
| SM/U/SEP20/31.2 | *Acinetobacter pitti* | S | S | S | S | S | S | S | S | - | S | S | S | S | S | I | S |
| SM/U/SEP20/32.2 | *Acinetobacter pitti* | S | S | S | S | S | S | S | S | - | S | S | S | S | S | I | S |
| SM/U/SEP20/38.2 | *Acinetobacter gyllenbergii* | S | S | S | S | S | S | S | S | - | S | S | S | S | S | I | S |
| SM/H/DEC20/5.1 | *Acinetobacter calcoaceticus* | S | S | S | S | S | S | S | S | - | S | S | S | S | S | I | S |
| SM/H/DEC20/6.1 | *Acinetobacter calcoaceticus* | S | S | S | S | S | S | S | S | - | S | S | S | S | S | I | S |
| SM/U/DEC20/21.3 | *Acinetobacter pitti* | R | R | R | I | R | R | R | R | - | R | S | S | S | S | I | S |
| SM/U/DEC20/22.6 | *Acinetobacter lwoffii* | - | S | - | S | - | S | - | S | - | S | - | S | - | S | - | S |
| SM/U/DEC20/28.5 | *Acinetobacter pitti* | S | S | S | S | S | S | S | S | - | S | S | S | S | S | I | S |
| SM/U/DEC20/29.2 | *Acinetobacter pitti* | S | R | S | S | S | S | S | S | - | S | S | S | S | S | I | S |
| SM/U/DEC20/29.5 | *Acinetobacter pitti* | S | S | S | S | S | S | S | S | - | S | S | S | S | S | I | S |
| SM/U/DEC20/32.1 | *Acinetobacter pitti* | S | S | S | S | S | S | S | S | - | S | S | S | S | S | I | S |
| SM/U/DEC20/35.2 | *Acinetobacter pitti* | S | S | S | S | S | S | S | S | - | S | S | S | S | S | I | S |
| SM/U/DEC20/40.4 | *Acinetobacter calcoaceticus* | S | S | S | S | S | S | S | S | - | S | S | S | S | S | I | S |
| SM/H/APR21/2.2 | *Acinetobacter pitti* | S | S | S | S | S | S | S | S | - | S | S | S | S | S | I | S |
| SM/H/APR21/8.1 | *Acinetobacter junii* | S | S | S | S | S | S | S | S | - | S | S | S | S | S | I | S |
| SM/H/APR21/12.2 | *Acinetobacter pitti* | S | S | S | S | S | S | S | S | - | S | S | S | S | S | I | S |
| SM/H/APR21/20.1 | *Acinetobacter radioresistans* | S | S | S | S | S | S | S | S | - | S | S | S | S | S | I | S |
| SM/U/APR21/36.2 | *Acinetobacter pitti* | S | S | S | S | S | S | S | S | - | S | S | S | S | S | I | S |
| SM/U/APR21/38.2 | *Acinetobacter radioresistans* | S | S | S | S | S | S | S | S | - | S | S | S | S | S | I | S |
| SM/U/APR21/38.3 | *Acinetobacter radioresistans* | S | S | S | S | S | S | S | S | - | S | S | S | S | S | I | S |
| SM/U/APR21/40.2 | *Acinetobacter pitti* | S | S | S | S | S | S | S | S | - | S | S | S | S | S | I | S |

H, Hospital; U, University; S, susceptible; I, intermediate; R, resistant. “-“ denotes not applicable. TIC, Ticarcillin-clavulanic acid; PIT, Piperacillin tazobactam, CAZ, Ceftazidime; SCF, Cefeperozone-sulbactam; CEF, Cefeperazone; DOR, Doripenem,; IMP, Imipenem; MRP, Meropenem; AK, Amikacin; GEN, Gentamycin; CIP, Ciprofloxacin; LEV, Levofloxacin; MIN, Minocycline; TGC, Tigecycline; COT, Trimethoprim-sulphamethoxazole. CL, Colistin.

Supplementary Table 3

**Table 3:** **AST pattern of the Klebsiella and Escherichia coli isolates obtained from each of the soil samples from JSS Hospital and JSS University**.

| **SAMPLE ID** | **ORGANISM** | **AMP** | **AMC** | **PIT** | **CXM** | **CXM /**  **AXT** | **CTX** | **SCF** | **CPM** | **ETP** | **IPM** | **MRP** | **AK** | **GEN** | **NA** | **CIP** | **TGC** | **NIT** | **CL** | **COT** |
| --- | --- | --- | --- | --- | --- | --- | --- | --- | --- | --- | --- | --- | --- | --- | --- | --- | --- | --- | --- | --- |
| SM/H/SEP20/2.1 | *Klebsiella pneumoniae* | R | S | S | S | S | S | S | S | S | S | S | S | S | S | S | S | I | I | S |
| SM/H/SEP20/3.1 | *Klebsiella pneumoniae* | R | S | S | S | S | S | S | S | S | S | S | S | S | S | S | S | I | I | S |
| SM/H/SEP20/4.3 | *Klebsiella pneumoniae* | R | S | S | S | S | S | S | S | S | S | S | S | S | S | S | S | I | I | S |
| SM/H/SEP20/9.2 | *Klebsiella varicola* | R | S | S | S | S | S | S | S | S | S | S | S | S | S | S | S | I | I | S |
| SM/H/SEP20/10.1 | *Klebsiella pneumoniae* | R | S | S | S | S | S | S | S | S | S | S | S | S | S | S | S | I | I | S |
| SM/H/AUG20/13.2 | *Klebsiella pneumoniae* | R | S | S | S | S | S | S | S | S | S | S | S | S | S | S | S | I | I | S |
| SM/H/SEP20/19.2 | *Klebsiella pneumoniae* | R | S | S | S | S | S | S | S | S | S | S | S | S | S | S | S | I | I | S |
| SM/U/SEP20/30.1 | *Klebsiella oxytoca* | R | S | S | S | S | S | S | S | S | S | S | S | S | S | S | S | I | I | S |
| SM/U/SEP20/31.3 | *Klebsiella oxytoca* | R | S | S | S | S | S | S | S | S | S | S | S | S | S | S | S | S | I | S |
| SM/U/SEP20/32.3 | *Klebsiella oxytoca* | R | S | S | S | S | S | S | S | S | S | S | S | S | S | S | S | S | I | S |
| SM/U/SEP20/36.1 | *Klebsiella oxytoca* | R | R | S | S | S | S | S | S | S | S | S | S | S | S | S | S | S | I | S |
| SM/U/SEP20/37.2 | *Klebsiella pneumoniae* | R | S | S | S | S | S | S | S | S | S | S | S | S | S | S | S | S | I | S |
| SM/U/SEP20/37.3 | *Klebsiella pneumoniae* | R | R | S | S | S | S | S | S | S | S | S | S | S | S | S | S | I | I | S |
| SM/H/DEC20/20.4 | *Klebsiella pneumoniae* | R | S | S | S | S | S | S | S | S | S | S | S | S | S | S | S | I | I | S |
| SM/U/DEC20/39.2 | *Klebsiella pneumoniae* | R | S | S | S | S | S | S | S | S | S | S | S | S | S | S | S | I | I | S |
| SM/H/SEP20/3.2 | *Escherichia coli* | S | S | S | S | S | S | S | S | S | S | S | S | S | S | S | S | S | I | S |
| SM/H/SEP20/4.1 | *Escherichia coli* | S | S | S | S | S | S | S | S | S | S | S | S | S | S | S | S | S | I | S |
| SM/H/SEP20/11.3 | *Escherichia coli* | S | S | S | S | S | S | S | S | S | S | S | S | S | S | S | S | S | I | S |
| SM/U/SEP20/30.4 | *Escherichia coli* | S | S | S | S | S | S | S | S | S | S | S | S | S | S | S | S | S | I | S |
| SM/U/SEP20/36.3 | *Escherichia coli* | S | S | S | S | S | S | S | S | S | S | S | S | S | S | S | S | S | I | S |
| SM/U/SEP20/38.1 | *Escherichia coli* | R | S | S | R | R | R | S | S | S | S | S | S | S | S | S | S | S | I | S |
| SM/U/SEP20/40.1 | *Escherichia coli* | S | S | S | S | S | S | S | S | S | S | S | S | S | S | S | S | S | I | S |
| SM/U/DEC20/28.1 | *Escherichia coli* | R | S | S | R | R | R | S | S | S | S | S | S | S | S | S | S | R | I | S |
| SM/U/DEC20/38.1 | *Escherichia coli* | S | S | S | S | S | S | S | S | S | S | S | S | S | S | S | S | S | I | S |
| SM/U/APR21/30.4 | *Escherichia coli* | S | S | S | S | S | S | S | S | S | S | S | S | S | S | S | S | S | I | S |

H, Hospital; U, University; S, susceptible; I, intermediate; R, resistant. “-“ denotes not applicable. AMP, Ampicillin; AMC, Amoxyclav; TIC, Ticarcillin-clavulanic acid; PIT, Piperacillin tazobactam, CXM, Cefuroxime; CXM/AXT, Cefuroxime auxetil; CTX, Ceftriaxone; CAZ, Ceftazidime; SCF, Cefeperozone-sulbactam; CEF, Cefeperazone; CPM, Cefepime; DOR, Doripenem,; IMP, Imipenem; MRP, Meropenem; ETP, Ertapenem; AK, Amikacin; GEN, Gentamycin; CIP, Ciprofloxacin; LEV, Levofloxacin; MIN, Minocycline; TGC, Tigecycline; COT, Trimethoprim-sulphamethoxazole. CL, Colistin; NIT, Nitrofurantoin; NA, Nalidixic acid.
